# Preventable causes of cancer in Texas by race/ethnicity: Major modifiable risk factors in the population

**DOI:** 10.1371/journal.pone.0274905

**Published:** 2022-10-13

**Authors:** Franciska J. Gudenkauf, Aaron P. Thrift

**Affiliations:** 1 Department of Medicine, Section of Epidemiology and Population Sciences, Baylor College of Medicine, Houston, Texas, United States of America; 2 University of Texas Health Science Center at Houston School of Public Health, Houston, Texas, United States of America; 3 Dan L Duncan Comprehensive Cancer Center, Baylor College of Medicine, Houston, Texas, United States of America; University of New South Wales, AUSTRALIA

## Abstract

**Background:**

A number of modifiable risk factors have been designated as being causally related to cancer development. We aimed to estimate the percentage of incident cancer cases diagnosed in persons aged ≥25 years in Texas in 2015, overall and by race/ethnicity, that were attributable to these modifiable risk factors.

**Methods:**

We calculated population attributable fractions (PAFs) for cancers attributable to thirteen modifiable risk factors using prevalence data from the Texas Behavioral Risk Factor Surveillance System and the National Health and Nutrition Examination Survey, as well as relative risks estimates from prior studies and cancer incidence data from the Texas Cancer Registry.

**Results:**

Overall, 32.3% of all incident cancers (N = 33,416) in 2015 were attributable to modifiable risk factors. Men (35.1%) had a numerically higher overall PAF than women (29.5%). Tobacco smoking caused the highest proportion of cancers (18.4%), followed by overweight and obesity (6.6%) and excess alcohol consumption (2.9%). Non-Hispanic Blacks had a numerically higher overall PAF (36.8%) than non-Hispanic Whites (31.9%) and Hispanics (31.7%). Further, non-Hispanic Blacks had the highest combined PAFs for 85% of cancer sites analyzed, including lung/bronchus and mouth/pharynx/larynx.

**Conclusion:**

Modifiable risk factors cause about one third of cancers in Texas. Non-Hispanic Blacks are especially affected by an excessive preventable cancer burden.

## Introduction

Through extensive epidemiological and experimental research, it is well established that cancer initiation and progression is driven by the interaction of host, environmental, and dietary or lifestyle factors [1]. Environmental, dietary, and lifestyle factors are potentially modifiable and are therefore the focus of population-level cancer prevention efforts.

The World Cancer Research Fund (WCRF)/American Institute for Cancer Research (AICR) and the International Agency for Research on Cancer (IARC) are dedicated to the systematic review and meta-analysis of global research on cancer prevention, and to the identification of causal relationships between risk factors and cancer development [1,2]. Through their Continuous Update Project and specifically their 2018 Third Expert Report, WCRF/AICR has classified many modifiable risk factors as cancer-causative or cancer-protective [1]. IARC has also determined the carcinogenic status of many risk factors through its *Monographs Programme* [2]. Understanding the burden of cancer caused by modifiable risk factors is critical for the prioritization of primary prevention efforts. Further, cancer incidence rates in the United States vary widely among different racial/ethnic subgroups [3]. Yet, it is not clear how the cancer burden attributable to modifiable risk factors differs by race/ethnicity. Identifying these differences may be useful for tailoring cancer prevention recommendations, which may ultimately help achieve cancer health equity.

We therefore aimed to estimate the population attributable fractions (PAFs) of cancer cases diagnosed in Texas in 2015 that were attributable to modifiable risk factors, overall as well as by race/ethnicity. We included in this analysis modifiable risk factors for which there is either “strong” evidence according to WCRF/AICR (i.e., there is either a “convincing” or “probable” causal relationship) [1] or “sufficient” evidence according to IARC (i.e., Group 1 or Group 2A level evidence) [2] of a causal relationship between the risk factor and cancer development. Modifiable risk factors were included if relevant to cancer burden in our chosen population, and if there are currently available population databases surveilling the risk factor. Specifically, we analyzed cancers attributable to tobacco smoking, overweight and obesity, excess alcohol consumption, insufficient physical activity, oncogenic infections (human papillomavirus [HPV] infection, chronic hepatitis C virus [HCV] infection, chronic *Helicobacter pylori* infection, chronic hepatitis B virus [HBV] infection, human herpesvirus-8 [HHV-8] infection), and inadequate diet (insufficient fiber intake, processed meat consumption, insufficient calcium intake, and red meat consumption). Furthermore, we stratified our analysis by major racial/ethnic subgroups of the population to reveal any disparities in the cancer burden attributable to modifiable risk factors. We chose Texas as the setting for our study because the diversity of this minority-majority population allows for a unique opportunity to explore the role of race/ethnicity as an effect modifier in preventable cancer burden.

## Materials and methods

The Texas Cancer Registry provided counts of incident invasive cancers diagnosed in Texas in 2015, overall and by age group (25–34, 35–44, 45–54, 55–64, 65–74, 75–84, ≥85 years), sex, and race/ethnicity (non-Hispanic Whites, non-Hispanic Blacks, Hispanics, Other Races/Ethnicities) [4]. We identified cancer sites of interest using a combination of International Classification of Diseases (ICD)-O-3 site codes and histology codes [5]. Some cancer sites were grouped depending on available risk estimates or WCRF/AICR or IARC conclusions; for example, mouth, pharyngeal, and laryngeal cancers were grouped together into one site.

After identifying the modifiable risk factors for which there is either “strong” evidence according to WCRF/AICR or “sufficient” evidence according to IARC, relative risk (RR) estimates for each modifiable risk factor were taken directly from the WCRF/AICR’s Third Expert Report if available [1], or from similar high-quality publications of large epidemiologic studies. We used sex-specific RRs where appropriate. We were unable to consider some risk factors due to lack of representative exposure data or because we had a lack of adequate occurrence data for the associated cancer type. S1 Table summarizes the cancers associated with each of the included modifiable risk factors and the associated relative risks that were used in the current analysis.

Depending on availability, we obtained weighted prevalence estimates of each modifiable risk factor from the Texas Behavioral Risk Factor Surveillance System (BRFSS) [6,7] or the National Health and Nutrition Examination Survey (NHANES) [8–11]. Consistent with prior PAF studies, we used prevalence estimates from the year 2006 to reflect a ten-year latency period between exposure and cancer diagnosis [12]. There were some exceptions. As BRFSS did not provide quantified physical activity estimates for the year 2006, we used 2005 data [7]. For dietary risk factors and oncogenic infections, we used NHANES Dietary Data [8] and Laboratory Data [9], respectively, from survey years 2005–2006 and assumed national estimates were broadly representative of exposure in Texas. For meat consumption, as the 2005–2006 survey data were not specific to red and processed meat, and patterns of meat consumption in the U.S. have not changed significantly [13], we used 2009–2010 data as an estimate of prevalence in 2005–2006 [10]. For *H*. *pylori* infection, prevalence data were only available from survey years 1999–2000 [11]; however, infection prevalence has not changed significantly from 1990 to 2006 [14], so we used this older data to estimate 2005–2006 prevalence. Information on derivation and categorization of risk factors are shown in Supplementary Materials. Exposure prevalence data are summarized in S2–S7 Tables.

Standard formulae were used to calculate PAFs for each modifiable risk factor by cancer site, age, sex, and race/ethnicity: $PAF=\frac{\sum \left(p_{x}*{ERR}_{x}\right)}{1+\sum \left(p_{x}*{ERR}_{x}\right)}$, where p_x_ is the population proportion at exposure category x (category of modifiable risk factor) and ERR_x_ is excess relative risk [15]. For most exposures, ${ERR}_{x}=({RR}_{x}-1)$ [15]; for alcohol consumption and dietary risk factors, ${ERR}_{x}=(e^{\left(Rg\times G_{x}\right)}-1)$ [16]. For detrimental exposures, Rg is increase in risk per unit consumption per day $\left(Rg=\frac{\ln\left(RR\right)}{y}\right)$, where y is the dose-response increment in units/day reported for that RR [16]. For protective exposures, Rg is increase in risk per unit deficit per day $\left(Rg=\frac{\ln\left(\frac{1}{RR}\right)}{y}\right)$ [17]. For detrimental exposures, G_x_ = (median consumption within exposure category x)–(reference level for exposure), and for protective exposures, G_x_ = (reference level for exposure)–(median consumption within exposure category x) [16]. HPV is a necessary cause of cervical cancer; its genome can be found in nearly all invasive carcinomas using the most sensitive methods of detection [18]. Likewise, HHV-8 is a necessary cause of Kaposi sarcoma. As such, and consistent with prior studies [18], we assumed the PAFs to be 100% for HPV-associated cervical cancer and HHV-8-associated Kaposi sarcoma.

PAFs were multiplied by incident cancer counts to obtain the number of excess cancer cases attributable to each modifiable risk factor, stratified by sex, age group, and race/ethnicity. The assumed ten-year latency period was accommodated by pairing prevalence estimates and derived PAFs in each age group with the cancer incidence age group ten years older (e.g., 2005 prevalence data for age group 25–34 years corresponded with 2015 cancer counts for age group 35–44 years). Age-weighted percentages of incident cancers attributable to each modifiable risk factor were calculated by cancer site, sex, and race/ethnicity; for cancers that were specified by histological subtype or sublocation (e.g., BMI-attributable esophageal adenocarcinoma), the number of attributable cancers of that specific histological subtype or sublocation was used as the numerator, while the overall cancer site incidence was used as the denominator (e.g., esophageal cancer incidence was used as the denominator for BMI-attributable esophageal adenocarcinoma). For each modifiable risk factor, all excess cases across all cancer sites were summed to estimate the percentage of all incident cancer cases (excluding basal cell carcinoma [BCC] and squamous cell carcinoma [SCC] of the skin) diagnosed in Texans aged ≥25 years in 2015 that were attributable to each modifiable risk factor.

We used previously described methods to calculate PAFs combining all risk factors [12,19]. For each cancer site, the total incident cancer count was multiplied by the greatest calculated PAF relevant to that cancer site to find the number of attributed cases to the risk factor associated with that PAF [12]. The attributed cases were then subtracted from the total incident cancer count to determine a new incident cancer count not yet attributed to a risk factor [12]. This new incident cancer count was then multiplied by the second greatest calculated PAF relevant to the cancer site, and so on for all relevant risk factors [12]. For all cancers overall, all risk factors were relevant. All attributable cases across all relevant risk factors were then summed and taken as a fraction of the total incident cancer count to find the combined PAF for all risk factors [12].

This study was conducted according to the guidelines laid down in the Declaration of Helsinki. All data were publically available. The research activities of this study were deemed as exempt by Baylor College of Medicine Ethics Committee as data were de-identified and aggregated. A waiver of consent was obtained because the study was a retrospective analysis and all data were aggregated and de-identified.

## Results

In 2015 in Texas, 103,408 cases of cancer (excluding BCC and SCC of the skin) were diagnosed in adults aged ≥25 years, of which 51,472 cases were in men and 51,936 cases were in women. Most cases occurred in non-Hispanic Whites (65,214), followed by Hispanics (22,642) and non-Hispanic Blacks (12,020). An additional 3,532 cases were diagnosed in individuals collectively grouped as being of other races/ethnicities.

**Fig 1:**
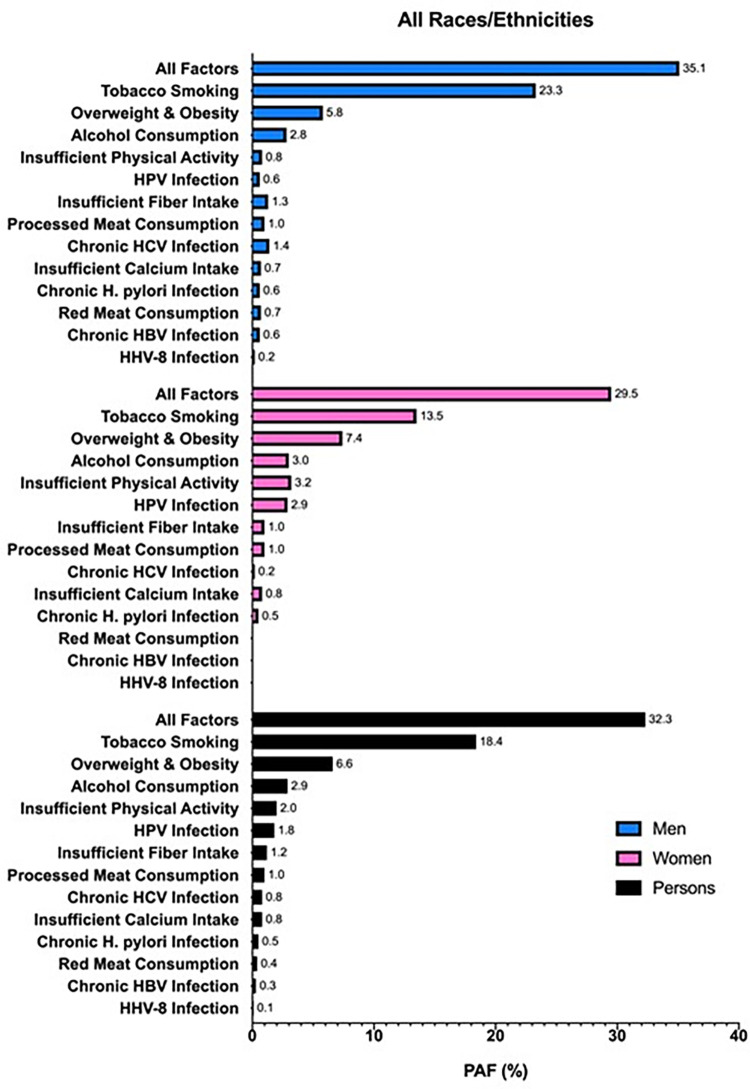
PAFs (%) of incident cancers attributable to modifiable risk factors in Texas in 2015 for men (blue), women (pink), and persons (black) of all races/ethnicities aged ≥25 years.

Overall, 32.3% of all new cancers (excluding BCC and SCC of the skin; N = 33,416) diagnosed in Texas in 2015 were attributable to the thirteen modifiable risk factors analyzed in this study (Fig 1). The risk factor causing the highest proportion of cancers was tobacco smoking (18.4%), followed by overweight and obesity (6.6%) and excess alcohol consumption (2.9%). A numerically greater overall percent of cancers was attributable to modifiable risk factors in men (35.1%) than in women (29.5%). In men, tobacco smoking (23.3%), overweight and obesity (5.8%), and excess alcohol consumption (2.8%) were the three risk factors with the highest overall PAFs; in women, tobacco smoking (13.5%), overweight and obesity (7.5%), and insufficient physical activity (3.2%) had the highest overall PAFs.

**Fig 2:**
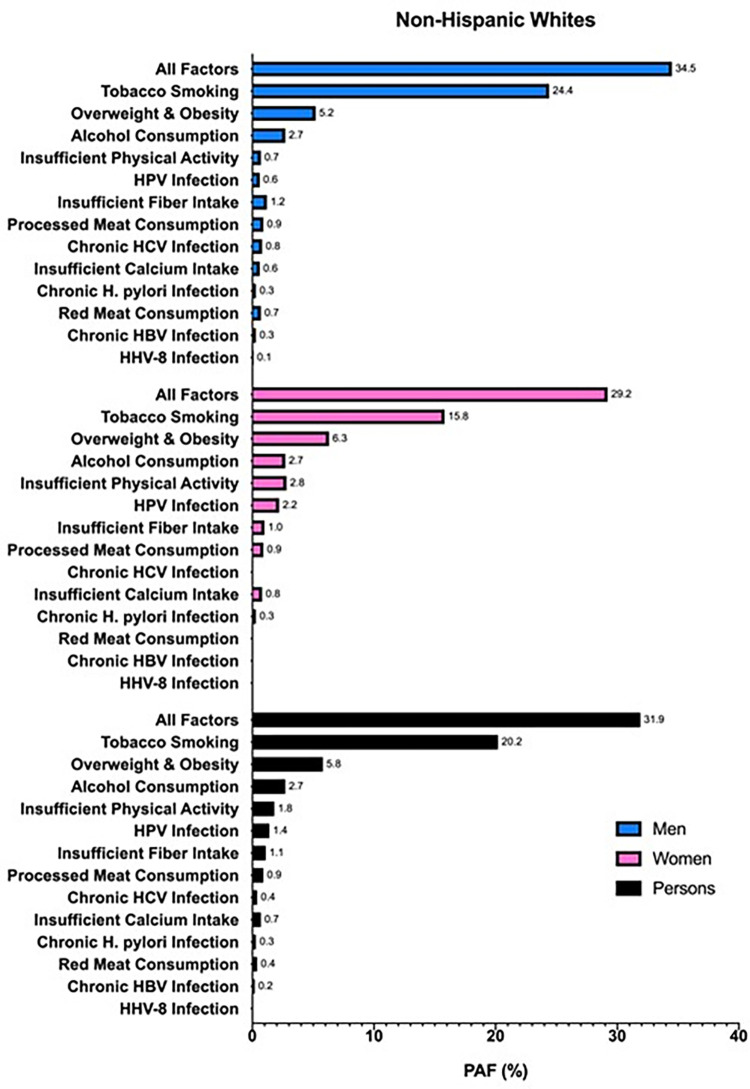
PAFs (%) of incident cancers attributable to modifiable risk factors in Texas in 2015 for non-Hispanic White men (blue), women (pink), and persons (black) aged ≥25 years.

**Fig 3:**
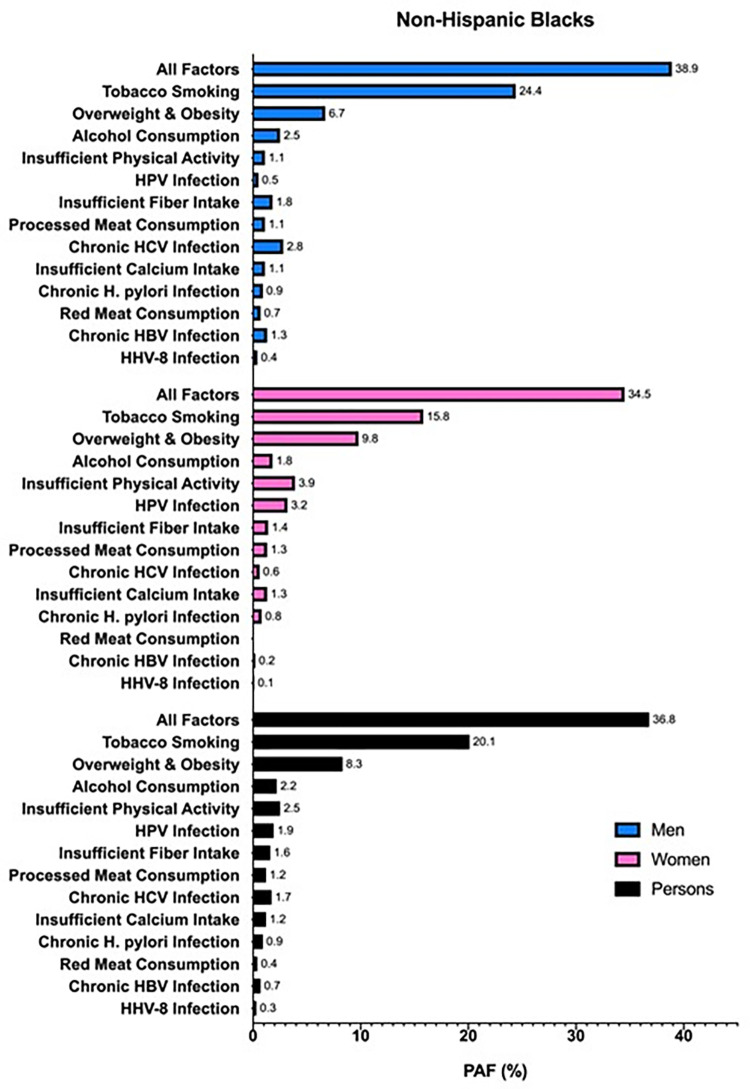
PAFs (%) of incident cancers attributable to modifiable risk factors in Texas in 2015 for non-Hispanic Black men (blue), women (pink), and persons (black) aged ≥25 years.

**Fig 4:**
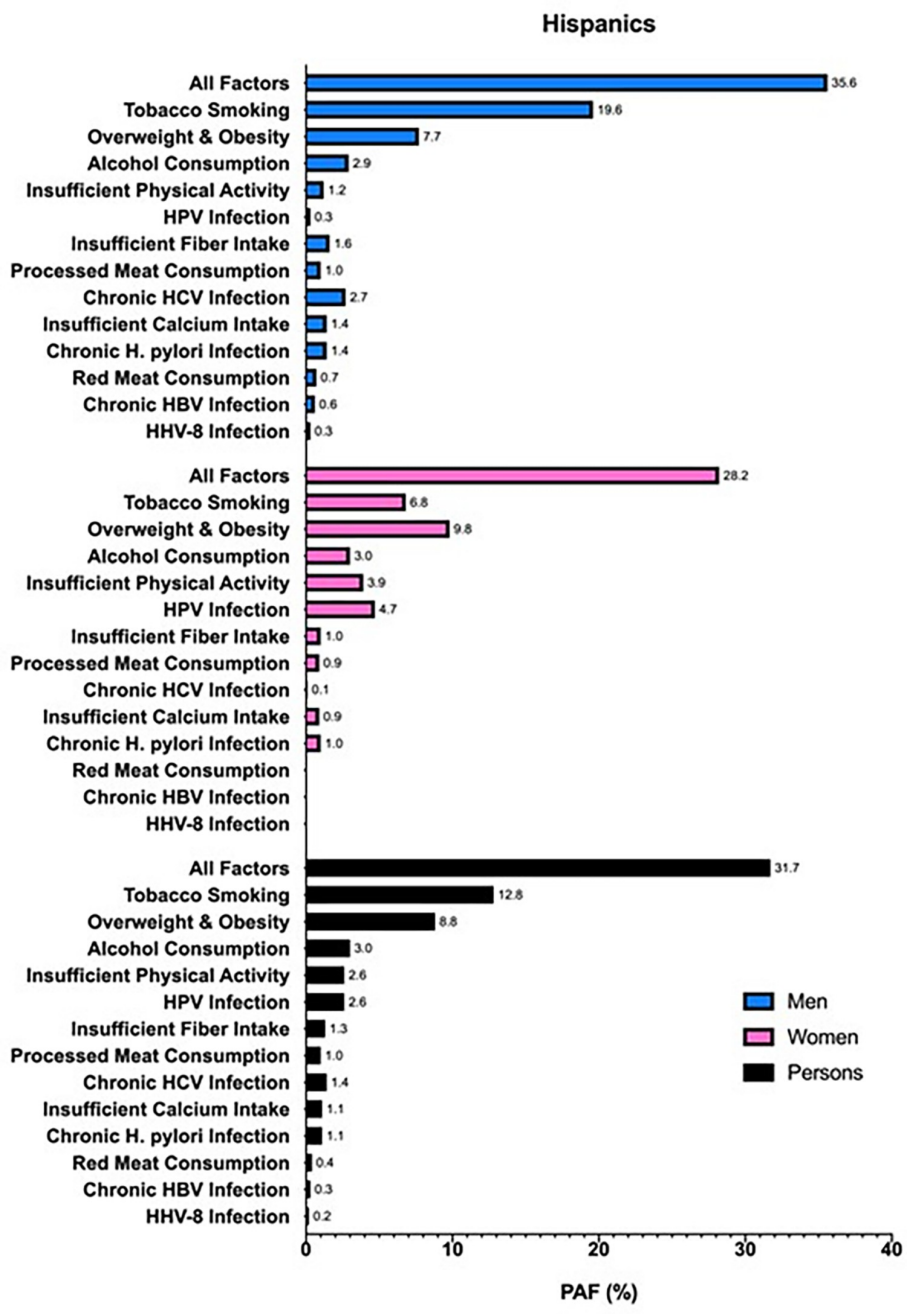
PAFs (%) of incident cancers attributable to modifiable risk factors in Texas in 2015 for Hispanic men (blue), women (pink), and persons (black) aged ≥25 years.

When stratified by race/ethnicity, 36.8% of cancers in non-Hispanic Blacks were attributable to modifiable risk factors, compared to 31.9% in non-Hispanic Whites and 31.7% in Hispanics (Figs 2–4). For cancers diagnosed among non-Hispanic Blacks, 20.1% were attributable to tobacco smoking, 8.3% to overweight and obesity, and 2.5% to insufficient physical activity; in Hispanics and non-Hispanic Whites, tobacco smoking (Hispanics, 12.8%; non-Hispanic Whites, 20.2%), overweight and obesity (Hispanics, 8.8%; non-Hispanic Whites, 5.8%), and excess alcohol consumption (Hispanics, 3.0%; non-Hispanic Whites, 2.7%) had the highest PAFs. Comparing each factor-specific PAF across the major racial/ethnic groups, non-Hispanic Blacks had numerically higher PAFs for six of the thirteen risk factors. Insufficient fiber intake, processed meat consumption, chronic HCV infection, insufficient calcium intake, chronic HBV infection, and HHV-8 infection caused more cancers in non-Hispanic Blacks than in Hispanics and non-Hispanic Whites; meanwhile, overweight and obesity, excess alcohol consumption, insufficient physical activity, HPV infection, and *H*. *pylori* infection caused more cancers in Hispanics, and tobacco smoking caused more cancers in non-Hispanic Whites than other racial/ethnic subgroups. There was no difference in overall PAF for red meat consumption across racial/ethnic groups. In men, tobacco smoking had the highest overall PAF across the major racial/ethnic subgroups, followed by overweight and obesity; however, the third highest overall PAF in non-Hispanic Black men was for HCV infection, as opposed to excess alcohol consumption in non-Hispanic White and Hispanic men. In women, tobacco smoking had the highest overall PAF in non-Hispanic Whites and non-Hispanic Blacks, followed by overweight and obesity and insufficient physical activity; yet, in Hispanic women, the highest ranked PAFs were for overweight and obesity, tobacco smoking, and then HPV infection.

**Fig 5:**
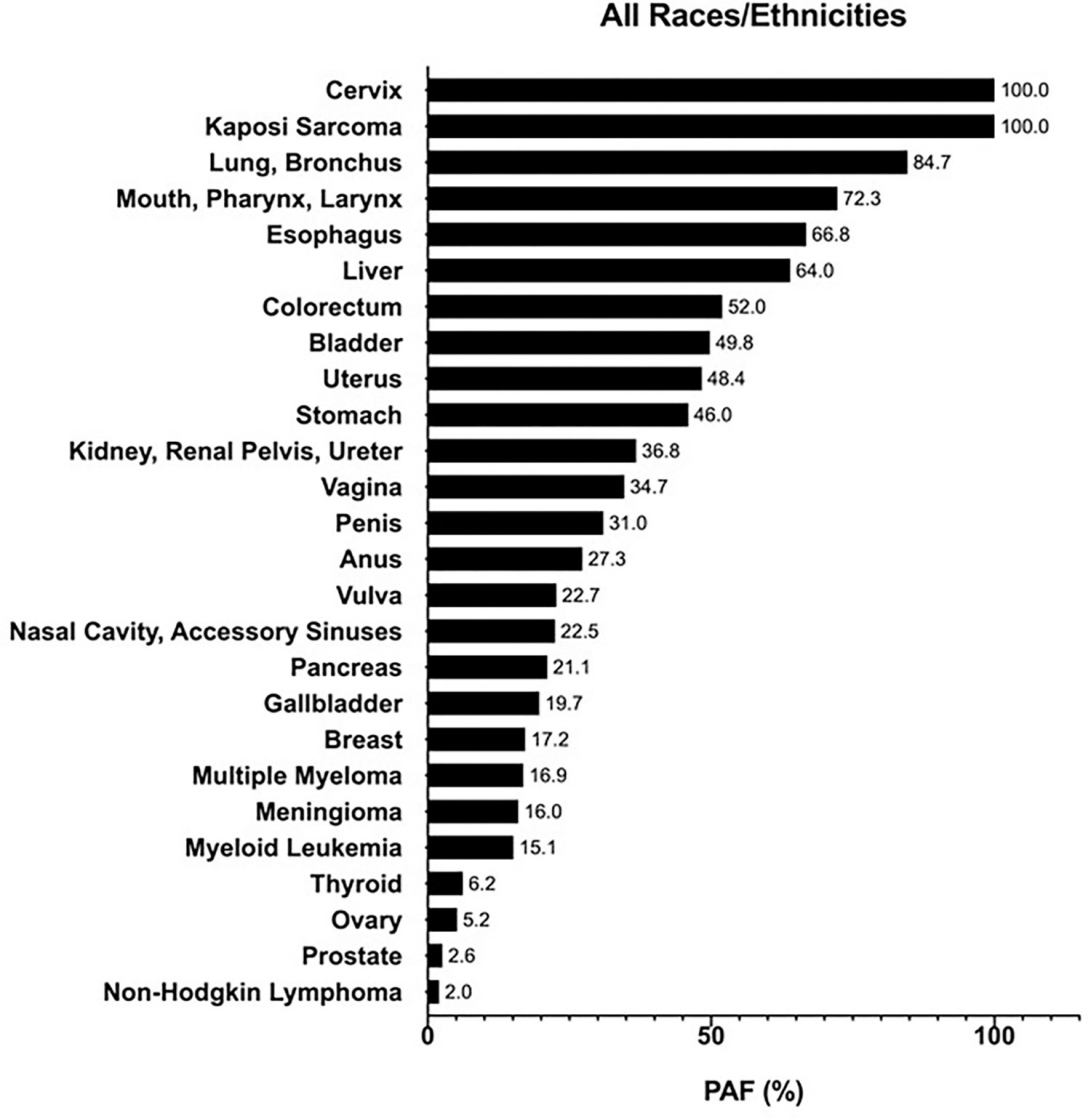
Combined PAFs (%) by cancer site for incident cancers attributable to all modifiable risk factors in Texas in 2015 for persons of all races/ethnicities aged ≥25 years.

For all risk factors combined, the cancer sites with the highest calculated PAFs (i.e., excluding cervix and Kaposi sarcoma, since PAFs were not calculated and assumed to be 100%) were lung and bronchus (84.7%); mouth, pharynx, and larynx (72.3%); and esophagus (66.8%) for all races/ethnicities combined (Fig 5). This trend was consistent in non-Hispanic Whites (lung, bronchus, 84.7%; mouth, pharynx, larynx, 71.0%; esophagus, 66.3%) and in Hispanics (lung, bronchus, 80.1%; mouth, pharynx, larynx, 69.8%; esophagus, 66.4%), while in non-Hispanic Blacks, lung and bronchus (87.5%); liver (79.4%); and mouth, pharynx, and larynx (77.8%) were the top three cancer sites with the highest PAFs for all risk factors combined (S1–S3 Figs). Comparing each cancer site across racial/ethnic groups, non-Hispanic Blacks had the highest site-specific PAFs at all but four of the twenty-six cancer sites: esophagus, breast, myeloid leukemia, and prostate–for which Hispanics had either a higher or equal PAF to non-Hispanic Blacks. Sex differences in site-specific PAFs overall and for each racial/ethnic group can be viewed in S4–S7 Figs, while S8–S12 Tables contain matrices of PAFs by cancer site and risk factor, overall and for each racial/ethnic subgroup.

## Discussion

In this analysis, we estimated that 32.3% of all cancers (excluding BCC and SCC of the skin) diagnosed in 2015 in Texans aged ≥25 years were attributable to modifiable risk factors, with a numerically greater proportion of attributable cancers in men (35.1%) than in women (29.5%). Tobacco smoking caused the highest proportion of cancers (18.4%) compared to the other risk factors analyzed, regardless of race/ethnicity. Non-Hispanic Blacks had a numerically higher overall proportion of cancers attributable to modifiable risk factors (36.8%) than non-Hispanic Whites (31.9%) and Hispanics (31.7%). When all risk factors were combined, the cancer site with the highest calculated PAF (i.e., excluding cervix and Kaposi sarcoma) was lung and bronchus, overall (84.7%) and for each racial/ethnic subgroup.

Our results contribute to the expanding literature on cancer burden attributable to preventable behaviors and environmental factors. A recent study in the U.S. estimated that 42.0% of cancers (excluding nonmelanoma skin cancers) in 2014 in American adults ≥30 years were attributable to potentially modifiable risk factors [20]. In Australia in 2010, 31.9% of all cancers were attributable to lifestyle and environmental factors [21]. In the United Kingdom in 2015, 37.7% of all cancers were attributable to modifiable risk factors [12], while in 2010, 42.7% of cancers were caused by lifestyle and environmental factors [19]. Although our results are comparable to these other studies, differences in risk factors and cancer sites considered, prevalence of risk factors, and overall methodology complicate direct comparisons in computed PAFs. Furthermore, the lower PAF from our Texas-based study (32.3%) compared to the U.S.-wide study (42.0%) may also reflect the different cancer burden in Texas (vs U.S.; less of the cancer types attributable to the risk factors examined) and the racial/ethnic distribution (with minority populations having higher exposure prevalence and higher burden of cancer types not attributable to the risk factors examined). Like our analysis, all of these other studies found that a higher proportion of cancers were preventable in men than in women (TX 2015: men, 35.1%; women, 29.5%. U.S. 2014 [20]: men, 42.5%; women, 41.5%. Australia 2010 [21]: men, 32.9%; women, 30.7%. U.K. 2015 [12]: men, 38.6%; women, 36.8%. U.K. 2010 [19]: men, 45.3%; women, 40.1%). The smaller difference between men and women in the U.S. study [20] again could reflect the pooling of data across geographic regions with known differences in cancer burden as well as lack of consideration for race/ethnic differences in risk factor prevalence and cancer burden.

Although similar studies have been carried out in the U.S. and other parts of the world, none have expounded the differences in cancer burden across major racial/ethnic subgroups of the population. We found that non-Hispanic Blacks had relatively more cancers caused by modifiable risk factors (36.8%) than non-Hispanic Whites (31.9%) and Hispanics (31.7%). Further, non-Hispanic Blacks also had more of the numerically highest factor-specific PAFs (46% of the risk factors) and site-specific PAFs (85% of the cancer sites) than either Hispanics or non-Hispanic Whites, which explains the higher overall PAF for all risk factors combined and all cancers. These findings have major public health implications as they suggest that, overall, non-Hispanic Blacks are disproportionately burdened by preventable cancers compared to other racial/ethnic subgroups. Yet, closer inspection of PAFs specific to sex, cancer site, and/or risk factor may suggest otherwise, thus emphasizing the importance of such a thorough and detailed investigation. For example, in Hispanic women, overweight and obesity contributes the highest proportion of the cancer burden compared to other risk factors, which is different than for women of other racial/ethnic subgroups, or even men. By appreciating which modifiable risk factors are most pertinent to cancer prevention at certain cancer sites for a specific racial/ethnic subgroup, this information may be strategically employed by cancer prevention programs in order to more effectively customize and prioritize prevention efforts. Ultimately, a more targeted approach may help achieve cancer health equity.

Our study has several limitations. Although our emphasis was on racial/ethnic effect modification, and we used race/ethnicity-specific prevalence data and incidence data, we were unable to use race/ethnicity-specific RR estimates, which may have influenced comparisons. Additionally, because we based our study in Texas, we attempted to use a state-level database for our prevalence estimates; however, when Texas-specific data were unavailable, we used NHANES data and assumed that these national data were representative of the Texas population. While it is possible that there are differences in the prevalence of infections and dietary risk factors from BRFSS and NHANES, this limitation does not impact the internal validity of our analysis, as estimates for each racial/ethnic subgroup came from the same data source. Other assumptions made throughout this analysis may also be considered limitations; for example, reference levels chosen may not actually represent the pathophysiological point of increased risk of cancer development, and latency periods may differ by cancer site and are not clearly established [12,15,19–21]. Additionally, while many of the studies sourced for RR estimates controlled for confounding factors, and while we used a method of combining PAFs that assumes risk factors are independent of each other and prevents overestimation by removing cancers already attributed to a risk factor from the running incidence, confounding potential may still remain [12,19]. However, in accordance with prior population-based studies [12,19–21], we did not omit individuals from this analysis in whom confounding characteristics may have been influential. Although the numbers of cancers attributable to the modifiable risk factors generated by these analyses appear precise, we remind readers that there is potential for error in these estimates due both to statistical uncertainty (precision) as well as variation in prevalence and risk estimates. We did not calculate confidence intervals for the PAF as there is no universally agreed approach. We likely underestimated the actual proportions of cancers attributable to some individual risk factors and all potentially modifiable factors combined as we did not include several other potentially modifiable risk factors because of a lack of representative exposure data. Finally, our category of Other Races/Ethnicities is small and simply aids in classifying individuals who do not identify with our chosen major racial/ethnic subgroups, and thus findings from this category may not represent other named racial/ethnic subgroups (for example, findings from this category may not apply to Asian Pacific Islanders).

In conclusion, we found that nearly one third of all cancers diagnosed in Texas in 2015 were attributable to modifiable risk factors, with a greater proportion of preventable cancers diagnosed in non-Hispanic Blacks than other racial/ethnic subgroups. Yet, given we were unable to include all potential preventable risk factors in the current analysis (e.g., we excluded UV radiation, reproductive factors, environmental toxins), our estimate of overall PAF is likely on the lower end of the preventable cancer burden in Texas. In general, greater prevention efforts focused on tobacco smoking, overweight/obesity, alcohol consumption, and physical activity would be invaluable to reducing cancer incidence and may also lessen long-standing racial/ethnic cancer disparities. While striving to achieve cancer health equity, cancer prevention programs should remain conscientious of racial/ethnic differences in cancers attributable to modifiable risk factors in order to more successfully prioritize and personalize primary cancer prevention.

## Supporting information

S1 Fig Combined PAFs (%) by cancer site for incident cancers attributable to all modifiable risk factors in Texas in 2015 for non-Hispanic White persons aged ≥25 years. (PPTX)

S2 Fig Combined PAFs (%) by cancer site for incident cancers attributable to all modifiable risk factors in Texas in 2015 for non-Hispanic Black persons aged ≥25 years. (PPTX)

S3 Fig Combined PAFs (%) by cancer site for incident cancers attributable to all modifiable risk factors in Texas in 2015 for Hispanic persons aged ≥25 years. (PPTX)

S4 Fig a. Combined PAFs (%) by cancer site for incident cancers attributable to all modifiable risk factors in Texas in 2015 for men of all races/ethnicities aged ≥25 years. b. Combined PAFs (%) by cancer site for incident cancers attributable to all modifiable risk factors in Texas in 2015 for women of all races/ethnicities aged ≥25 years.
(PPTX)

S5 Fig a. Combined PAFs (%) by cancer site for incident cancers attributable to all modifiable risk factors in Texas in 2015 for non-Hispanic White men aged ≥25 years. b. Combined PAFs (%) by cancer site for incident cancers attributable to all modifiable risk factors in Texas in 2015 for non-Hispanic White women aged ≥25 years.
(PPTX)

S6 Fig a. Combined PAFs (%) by cancer site for incident cancers attributable to all modifiable risk factors in Texas in 2015 for non-Hispanic Black men aged ≥25 years. b. Combined PAFs (%) by cancer site for incident cancers attributable to all modifiable risk factors in Texas in 2015 for non-Hispanic Black women aged ≥25 years.
(PPTX)

S7 Fig a. Combined PAFs (%) by cancer site for incident cancers attributable to all modifiable risk factors in Texas in 2015 for Hispanic men aged ≥25 years. b. Combined PAFs (%) by cancer site for incident cancers attributable to all modifiable risk factors in Texas in 2015 for Hispanic women aged ≥25 years.
(PPTX)

S1 Table Relative risks for the associations between evaluated risk factors and associated cancer types. (DOCX)

S2 Table Prevalence of alcohol consumption in Texans aged ≥18 years in 2006 (%), overall and by race/ethnicity and age group. (DOCX)

S3 Table Prevalence of tobacco smoking in Texans aged ≥18 years in 2006 (%), overall and by race/ethnicity and age group. (DOCX)

S4 Table Prevalence of Americans aged ≥18 years not meeting recommendations for red meat, processed meat, fiber, and calcium consumption (%), overall and by race/ethnicity. (DOCX)

S5 Table Prevalence (shown as percentage, %) of oncogenic infections by race/ethnicity in the U.S. in 2006 according to the National Health and Nutrition Examination Survey, in men, women, and persons aged ≥18 years. (DOCX)

S6 Table Prevalence of moderate- or vigorous-intensity physical activity in Texans aged ≥18 years in 2005 (%), overall and by race/ethnicity and age group. (DOCX)

S7 Table Prevalence of overweight and obesity among adults aged ≥18 years in Texas in 2006 (%), overall and by race/ethnicity and age group. (DOCX)

S8 Table Age-weighted PAFs of cancers attributable to modifiable risk factors in Texas in 2015 for all races/ethnicities (%), adults aged ≥25 years. (DOCX)

S9 Table Age-weighted PAFs of cancers attributable to modifiable risk factors in Texas in 2015 for non-Hispanic Whites (%), adults aged ≥25 years. (DOCX)

S10 Table Age-weighted PAFs of cancers attributable to modifiable risk factors in Texas in 2015 for non-Hispanic Blacks (%), adults aged ≥25 years. (DOCX)

S11 Table Age-weighted PAFs of cancers attributable to modifiable risk factors in Texas in 2015 for Hispanics (%), adults aged ≥25 years. (DOCX)

S12 Table Age-weighted PAFs of cancers attributable to modifiable risk factors in Texas in 2015 for Other Races/Ethnicities (%), adults aged ≥25 years. (DOCX)

S1 File (DOCX)
