# Supplementary figures and images for: Preventable causes of cancer in Texas by race/ethnicity: Major modifiable risk factors in the population
Source: PLoS One. 2022 Oct 13;17(10):e0274905. doi: 10.1371/journal.pone.0274905 (PMC9560474; doi:10.1371/journal.pone.0274905)

## Slide 1
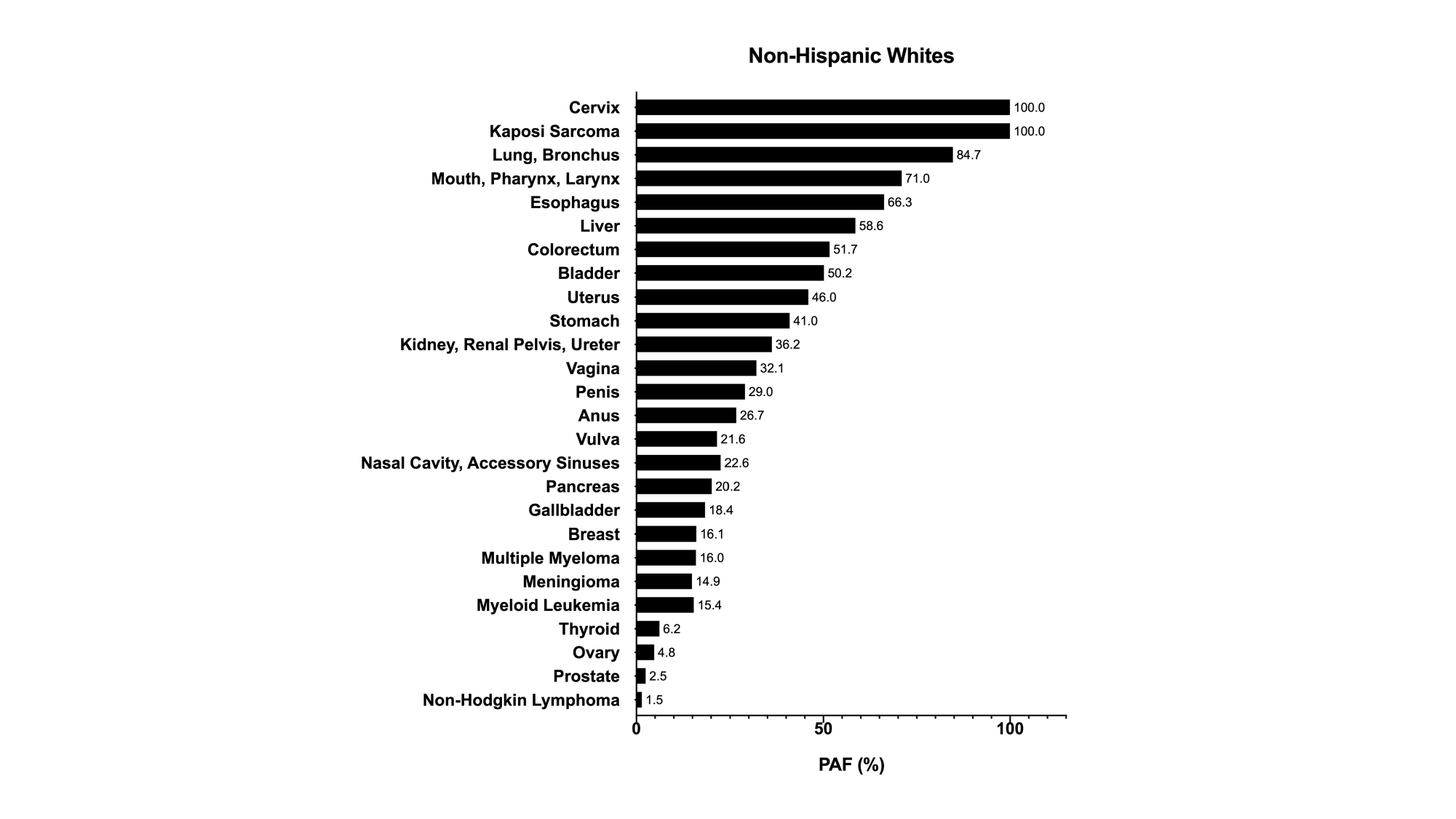

Supplement: S1 Fig — (PPTX) [file pone.0274905.s001.pptx]

## Slide 1
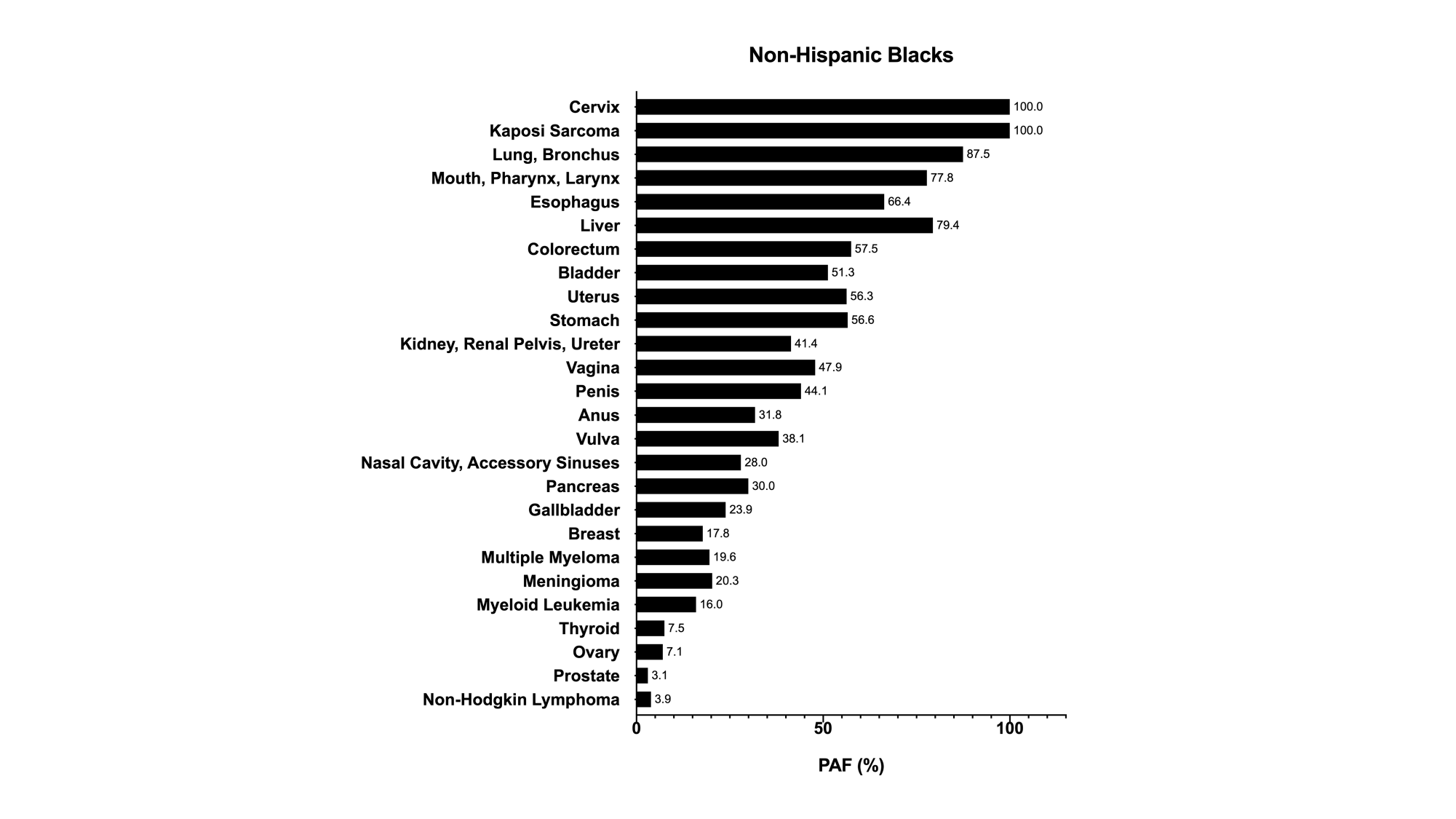

Supplement: S2 Fig — (PPTX) [file pone.0274905.s002.pptx]

## Slide 1
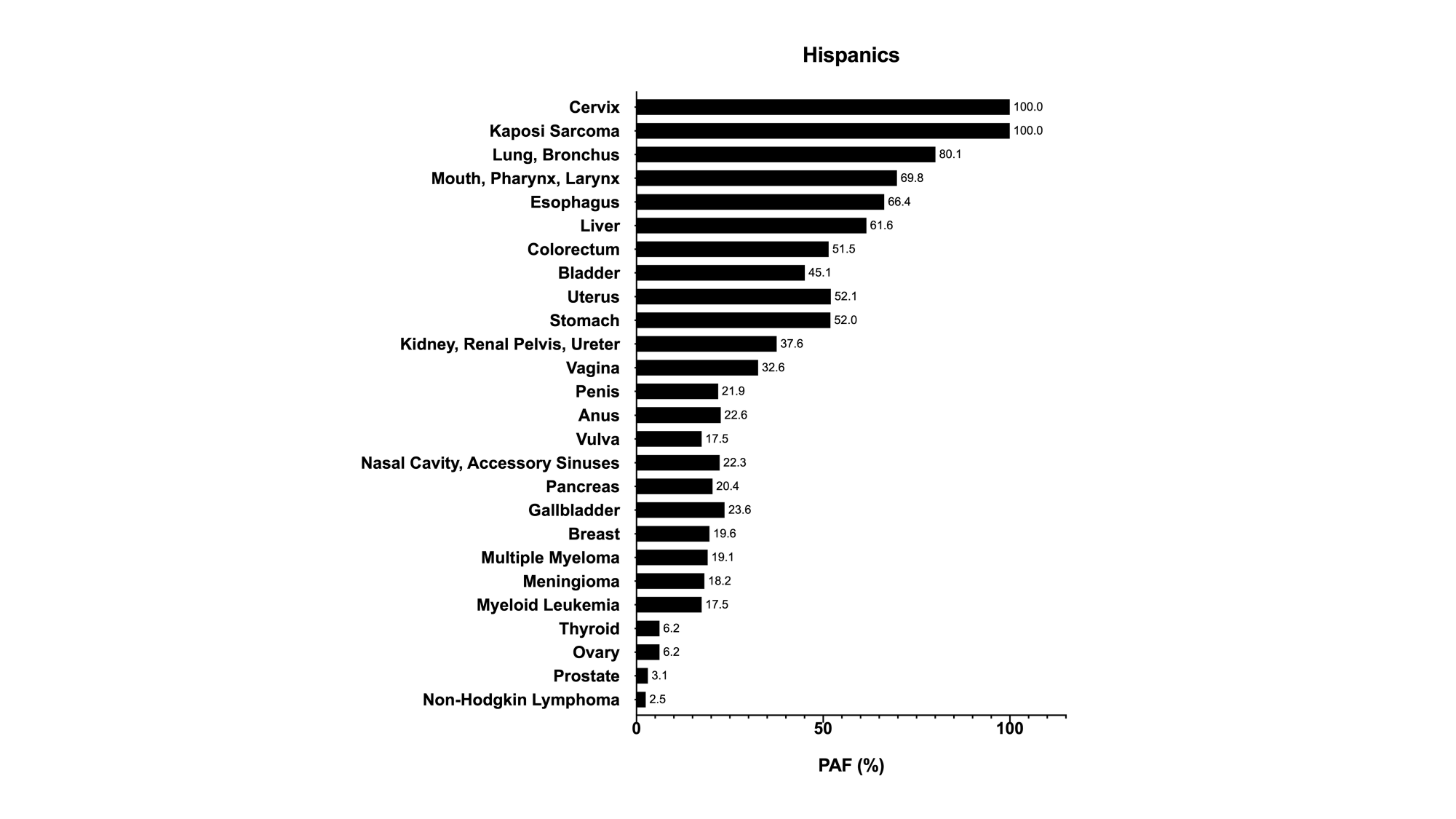

Supplement: S3 Fig — (PPTX) [file pone.0274905.s003.pptx]

## Slide 1
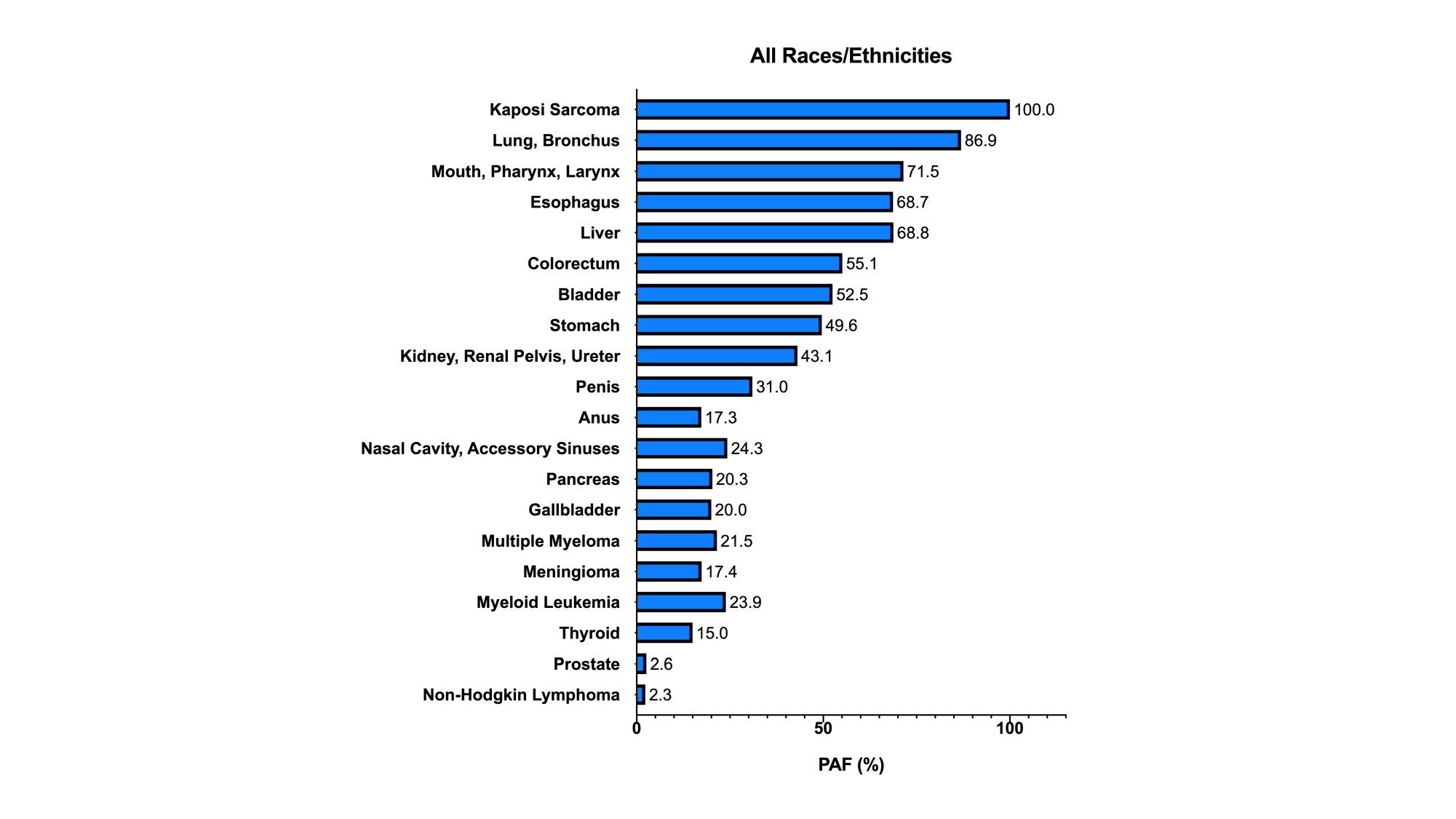

## Slide 2
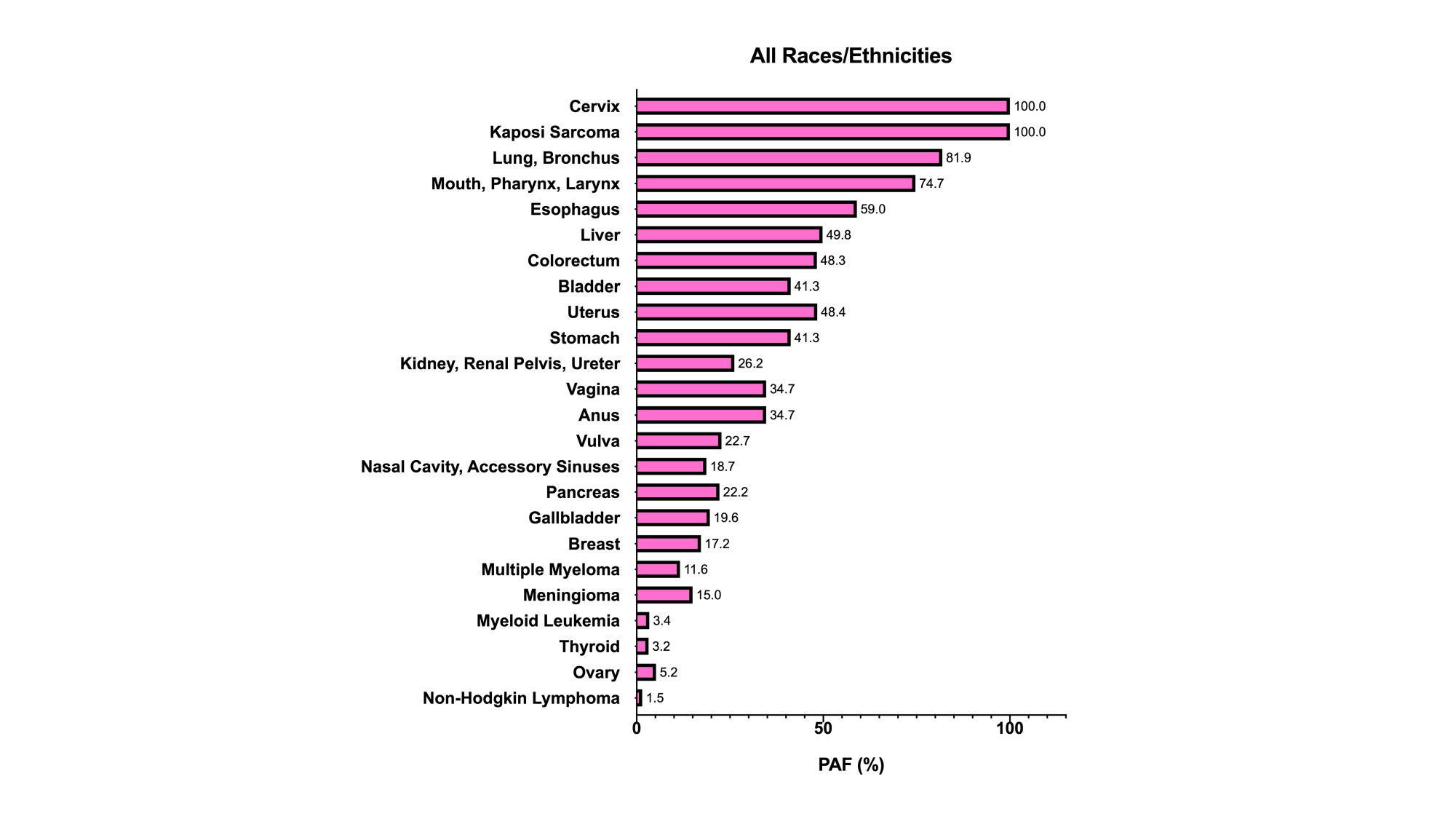

Supplement: S4 Fig — a. Combined PAFs (%) by cancer site for incident cancers attributable to all modifiable risk factors in Texas in 2015 for men of all races/ethnicities aged ≥25 years. b. Combined PAFs (%) by cancer site for incident cancers attributable to all modifiable risk factors in Texas in 2015 for women of all races/ethnicities aged ≥25 years. (PPTX) [file pone.0274905.s004.pptx]

## Slide 1
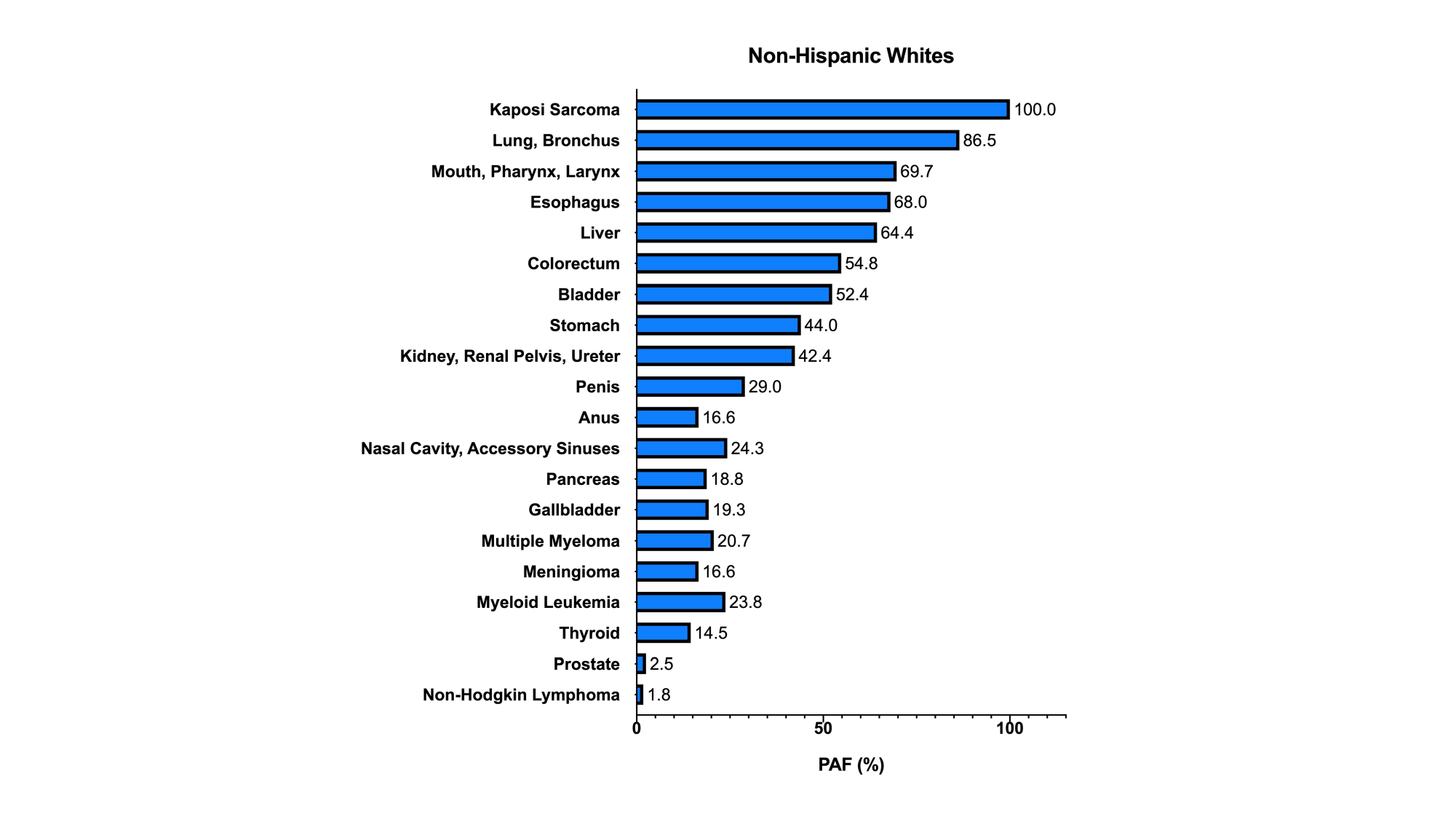

## Slide 2
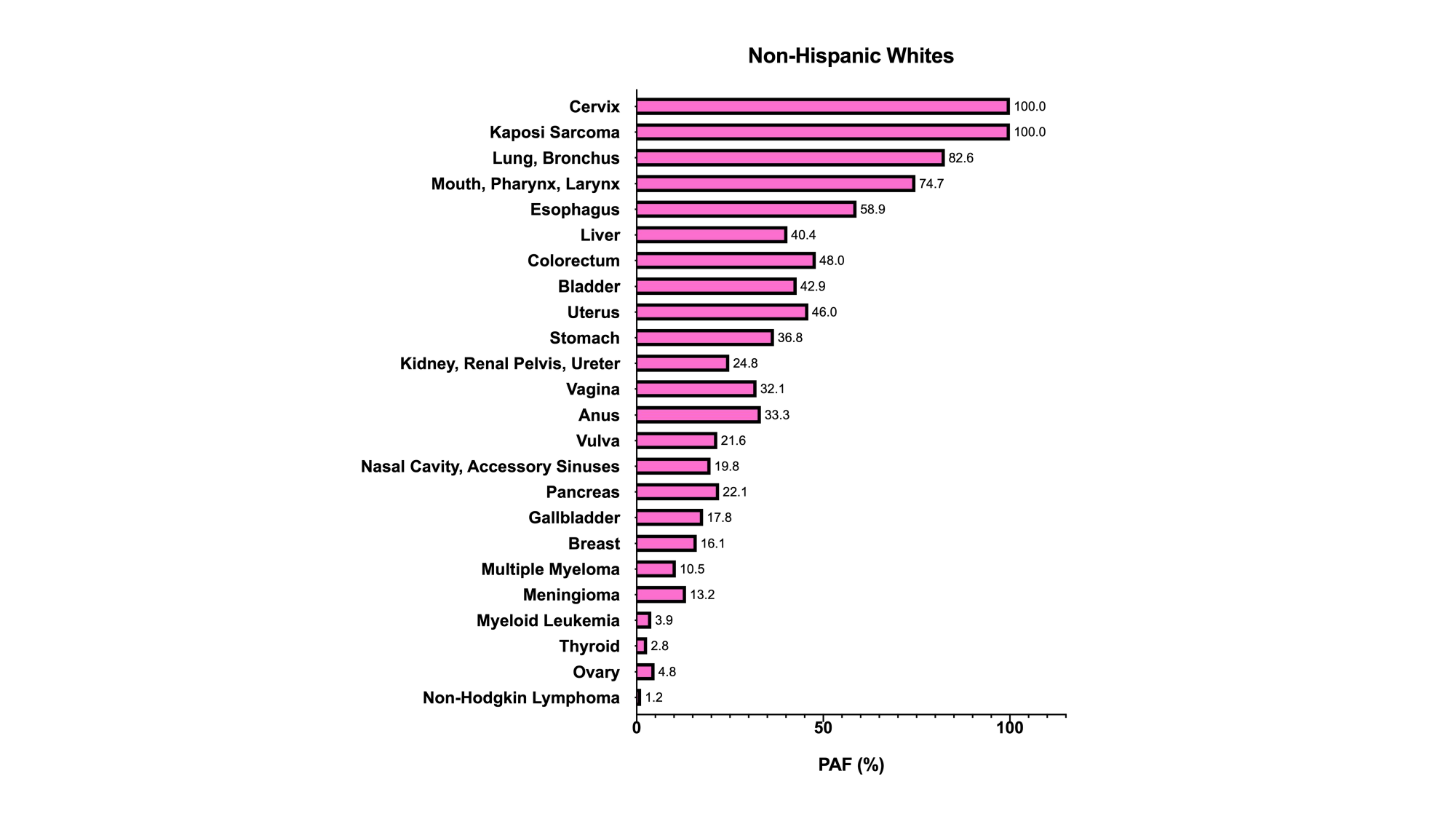

Supplement: S5 Fig — a. Combined PAFs (%) by cancer site for incident cancers attributable to all modifiable risk factors in Texas in 2015 for non-Hispanic White men aged ≥25 years. b. Combined PAFs (%) by cancer site for incident cancers attributable to all modifiable risk factors in Texas in 2015 for non-Hispanic White women aged ≥25 years. (PPTX) [file pone.0274905.s005.pptx]

## Slide 1
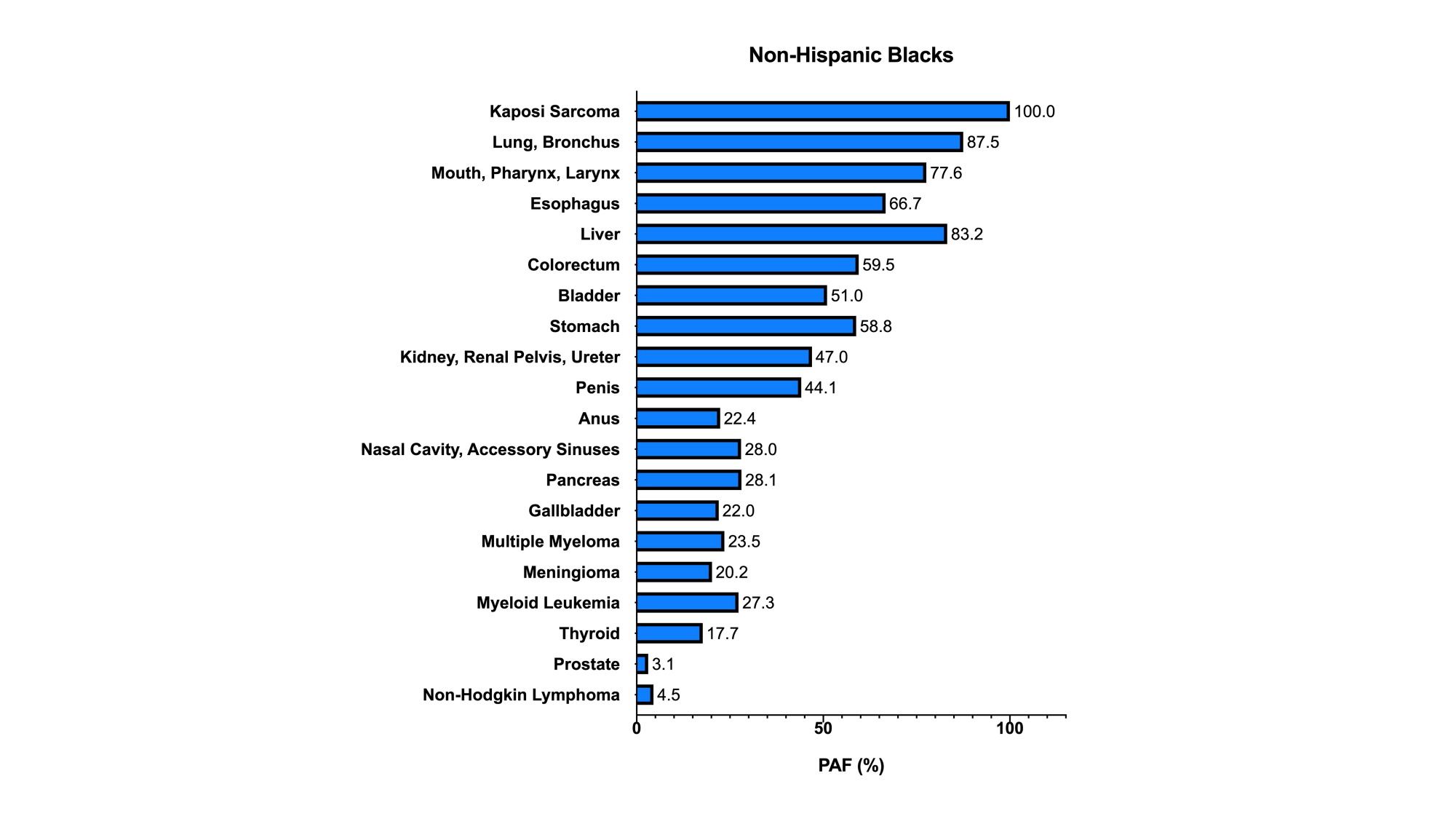

## Slide 2
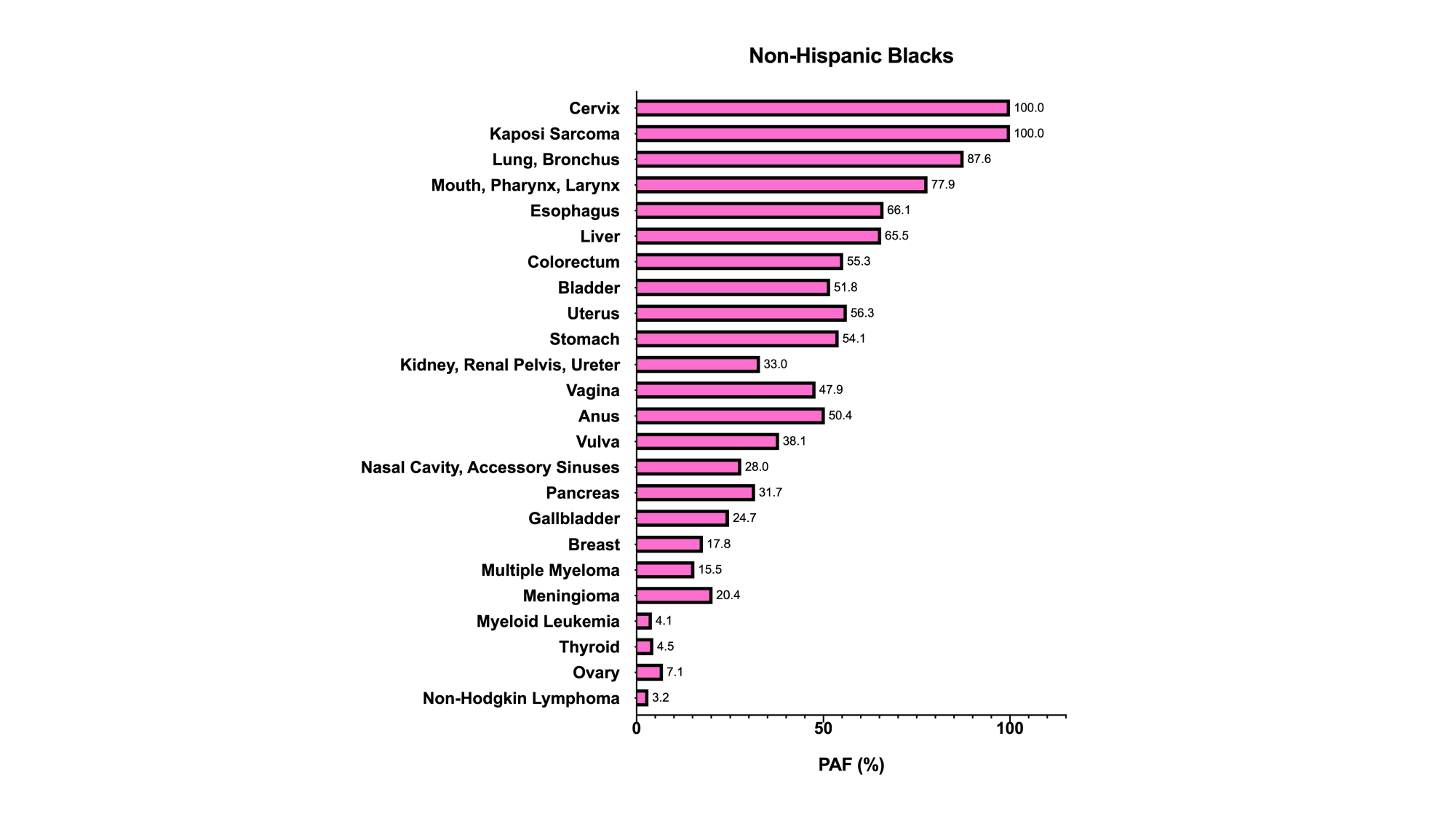

Supplement: S6 Fig — a. Combined PAFs (%) by cancer site for incident cancers attributable to all modifiable risk factors in Texas in 2015 for non-Hispanic Black men aged ≥25 years. b. Combined PAFs (%) by cancer site for incident cancers attributable to all modifiable risk factors in Texas in 2015 for non-Hispanic Black women aged ≥25 years. (PPTX) [file pone.0274905.s006.pptx]

## Slide 1
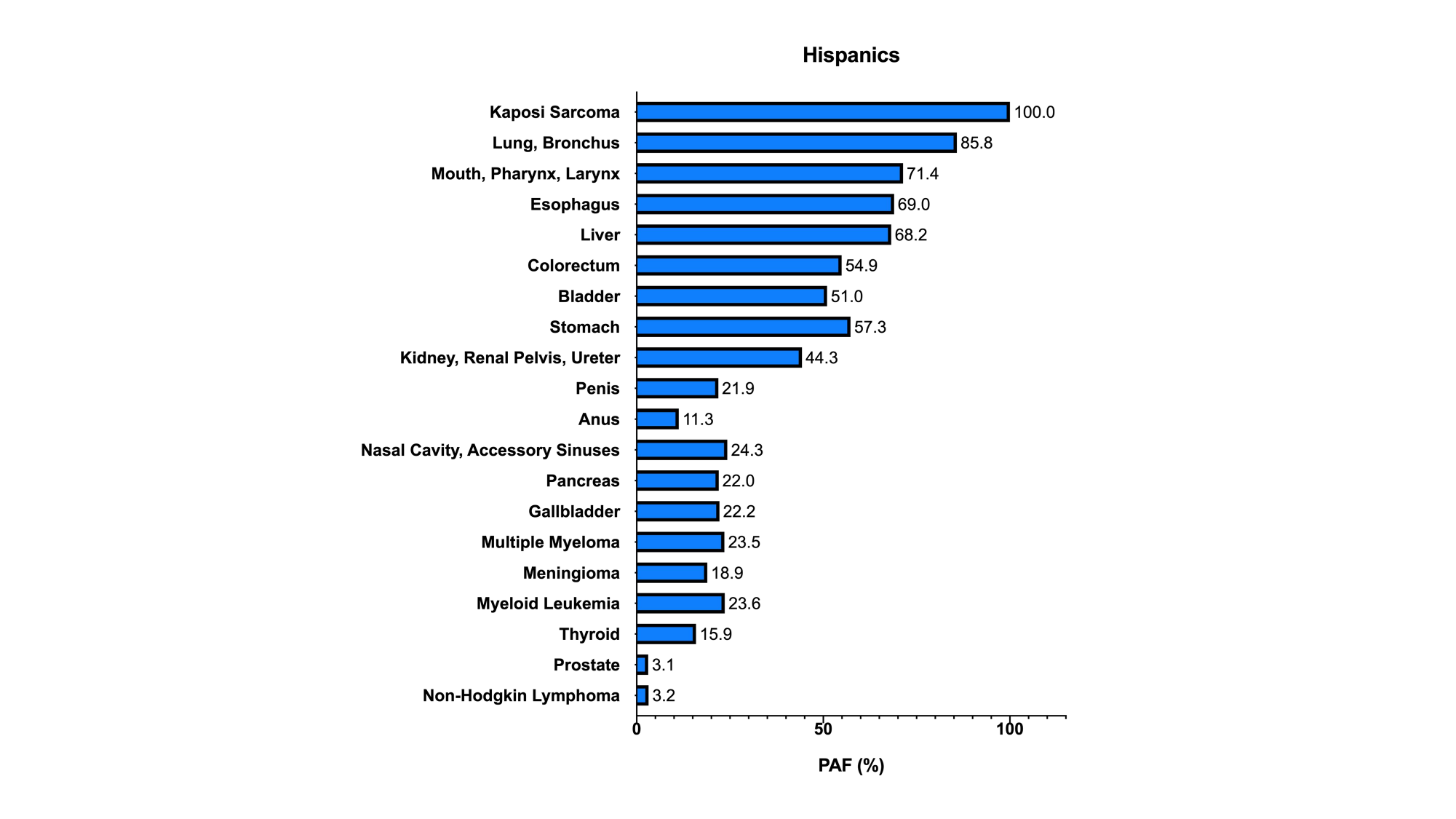

## Slide 2
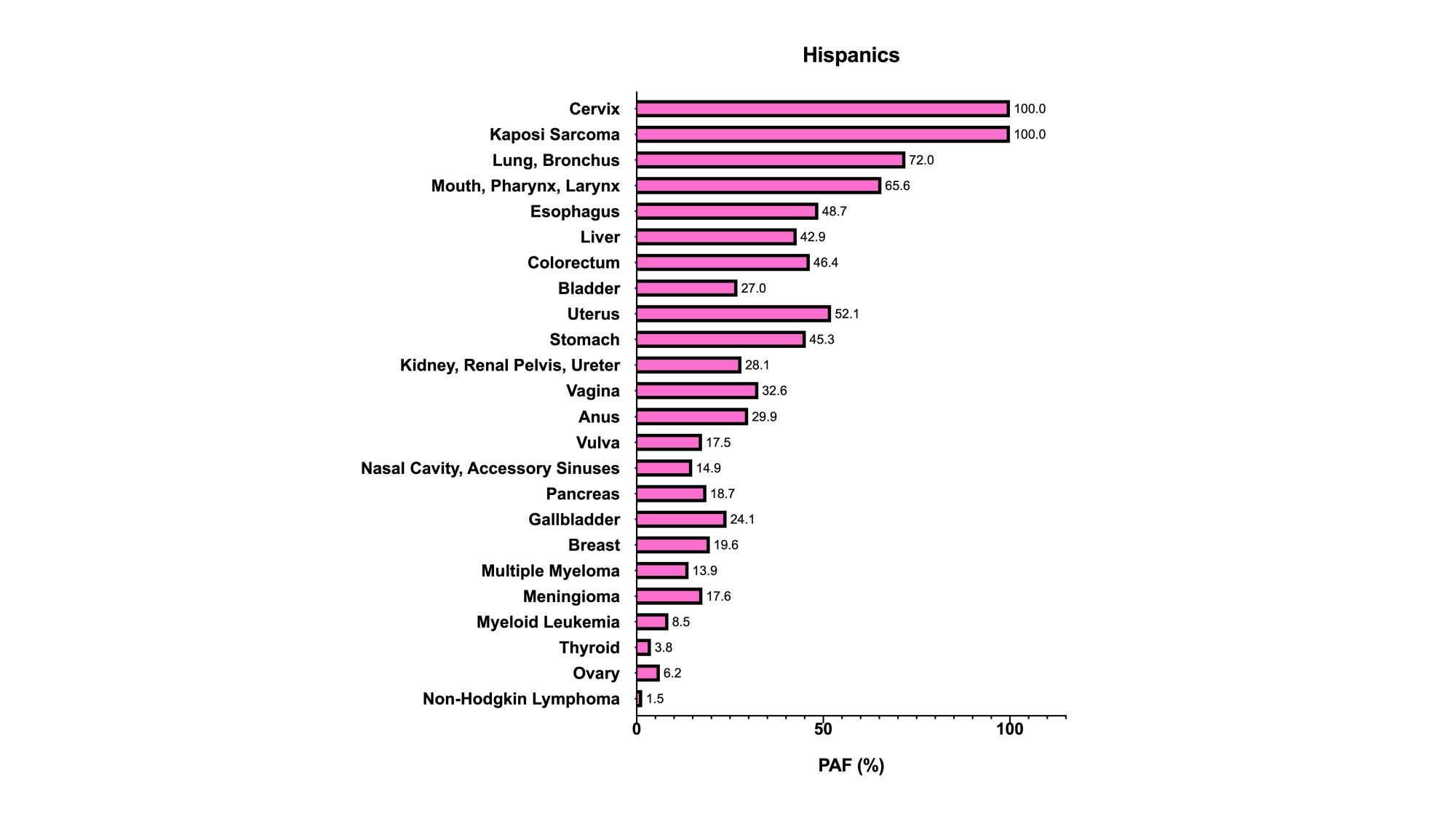

Supplement: S7 Fig — a. Combined PAFs (%) by cancer site for incident cancers attributable to all modifiable risk factors in Texas in 2015 for Hispanic men aged ≥25 years. b. Combined PAFs (%) by cancer site for incident cancers attributable to all modifiable risk factors in Texas in 2015 for Hispanic women aged ≥25 years. (PPTX) [file pone.0274905.s007.pptx]
