# Supplementary material for: Preventable causes of cancer in Texas by race/ethnicity: Major modifiable risk factors in the population
Source: PLoS One. 2022 Oct 13;17(10):e0274905. doi: 10.1371/journal.pone.0274905 (PMC9560474; doi:10.1371/journal.pone.0274905)
Supplement: S1 Table — (DOCX) [file pone.0274905.s008.docx]

**S1 Table.** Relative risks for the associations between evaluated risk factors and associated cancer types.

|  |  | **Relative Risk** | | |
| --- | --- | --- | --- | --- |
| **Cancer Site** | **Risk Measure** | **Men** | **Women** | **Persons** |
| **Alcohol intake** | **Daily intake** |  |  |  |
| Oral Cavity^1^ | Per 10 g/day | 1.13 | 1.24 | 1.15 |
|  | Rg (per 1g increase per day) | 0.0122 | 0.0215 | 0.0140 |
| Pharynx^1^ | Per 10 g/day | 1.11 | *1.25* | 1.13 |
|  | Rg (per 1g increase per day) | 0.0104 | 0.0223 | 0.0122 |
| Larynx^1^ | Per 10 g/day | 1.09 | 1.22 | 1.09 |
|  | Rg (per 1g increase per day) | 0.0086 | 0.0199 | 0.0086 |
| Liver^1^ | Per 10 g/day | 1.03 | 1.19 | 1.04 |
|  | Rg (per 1g increase per day) | 0.0030 | 0.0174 | 0.0039 |
| Colorectum^1^ | Per 10 g/day | 1.08 | *1.04* | 1.07 |
|  | Rg (per 1g increase per day) | 0.0077 | 0.0039 | 0.0068 |
| Stomach^1^ | Per 10 g/day | 1.03 | *1.02* | *1.02* |
|  | Rg (per 1g increase per day) | 0.0030 | 0.0020 | 0.0020 |
| Esophagus (squamous cell carcinoma)^1^  (Histology codes: 8050-8084) | Per 10 g/day | - | - | 1.25 |
|  | Rg (per 1g increase per day) | - | - | 0.0223 |
| Breast (pre-menopausal)^1^ | Per 10 g/day | - | 1.05 | - |
|  | Rg (per 1g increase per day) | - | 0.0049 | - |
| Breast (post-menopausal)^1^ | Per 10 g/day | - | 1.09 | - |
|  | Rg (per 1g increase per day) | - | 0.0086 | - |
| **Cigarette smoking** | **Smoking status (vs. never-smoking)** |  |  |  |
| Lung, bronchus^2,a^ | Current smoker | 25.3 | 22.9 |  |
|  | Former smoker | 6.8 | 6.8 |  |
| Oral cavity, pharynx^2,b^ | Current smoker | 5.7 | 5.6 |  |
|  | Former smoker | 1.7 | 2.2 |  |
| Larynx^2^ | Current smoker | 13.9 | 103.8 |  |
|  | Former smoker | 2.4 | 11.6 |  |
| Esophagus^2^ | Current smoker | 3.9 | 5.1 |  |
|  | Former smoker | 2.6 | 2.2 |  |
| Stomach^2^ | Current smoker | 1.9 | 1.7 |  |
|  | Former smoker | 1.5 | *1.1* |  |
| Pancreas^2^ | Current smoker | 1.6 | 1.9 |  |
|  | Former smoker | *1.0* | 1.2 |  |
| Colorectum^2^ | Current smoker | 1.4 | 1.6 |  |
|  | Former smoker | 1.2 | 1.2 |  |
| Liver^2^ | Current smoker | 2.3 | 1.8 |  |
|  | Former smoker | 1.5 | *1.1* |  |
| Kidney, renal pelvis, ureter^2,c^ | Current smoker | 1.8 | *1.2* |  |
|  | Former smoker | 1.5 | *1.2* |  |
| Urinary bladder^2^ | Current smoker | 3.9 | 3.9 |  |
|  | Former smoker | 2.4 | 2.3 |  |
| Cervix^3^ | Current smoker | - | 1.9 |  |
|  | Former smoker | - | 1.5 |  |
| Ovary (mucinous)^2,d^  (Histology codes: 8470,8471,8480,8481) | Current smoker | *-* | *1.1* |  |
|  | Former smoker | - | *1.1* |  |
| Myeloid leukemia^2,e^ | Current smoker | 1.9 | *1.1* |  |
|  | Former smoker | 1.4 | *1.1* |  |
| Nasal cavity, accessory sinuses^4,f^  (Primary site codes: C300,C310-319) | Current smoker | 1.95 | 1.95 |  |
|  | Former smoker | 1.39 | 1.39 |  |
| **Inadequate diet** |  |  |  |  |
| Red Meat^5^ | Per 100 g/day | *1.28* | *1.02* | *1.12* |
|  | Rg (per 1g increase per day)^g^ | 0.0025 | 0.0002 | 0.0011 |
| Processed Meat^5^ | Per 50 g/day | *1.11* | *1.18* | 1.16 |
|  | Rg (per 1g increase per day)^h^ | 0.0021 | 0.0033 | 0.0030 |
| Fiber^6^ | Per 10 g/day | 0.89 | 0.91 | *0.93* |
|  | Rg (per 1g deficit per day)^i^ | 0.0117 | 0.0094 | 0.0073 |
| Calcium^5^ | Per 200 mg/day | 0.93 | 0.93 | 0.94 |
|  | Rg (per 1mg deficit per day)^j^ | 0.0004 | 0.0004 | 0.0003 |
| **Human papillomavirus strain 16 (HPV-16)** |  |  |  |  |
| Vulva | HPV 16 L1 positive | — | 3.70 | — |
| Vagina | HPV 16 L1 positive | — | 6.30 | — |
| Penis | HPV 16 L1 positive | 11.60 | — | — |
| Anus | HPV 16 L1 positive | 5.30 | 5.90 | — |
| Oral cavity | HPV 16 L1 positive | — | — | 1.94 |
| Oropharynx, tonsil | HPV 16 L1 positive | — | — | 8.60 |
| Cervix^k,l^ | Not-applicable | NA^d^ | NA^d^ | NA^d^ |
| ***Heliobacter pylori*** |  |  |  |  |
| Stomach, noncardia (primary site codes: C161-166) | Anti-H. pylori IgG positive | — | — | 5.90 |
| Gastric MALToma^l^ (primary site codes: C160-169; histology code: 9699) | Anti-H. pylori IgG positive | — | — | 7.20 |
| **Hepatitis C virus** |  |  |  |  |
| Hepatocellular carcinoma (histology codes: 8170-8175) | Anti-HCV positive | — | — | 27.60 |
| Non-Hodgkin lymphoma | Anti-HCV positive | — | — | 1.78 |
| **Hepatitis B virus** |  |  |  |  |
| Hepatocellular carcinoma (histology codes: 8170-8175) | HbsAg positive | — | — | 23.40 |
| **Human herpesvirus type 8** |  |  |  |  |
| Kaposi sarcoma^l^ | Not-applicable | NA^m^ | NA^m^ | NA^m^ |
| **Moderate- or vigorous-intensity physical activity^n^** |  |  |  |  |
| Colon^3^ | 750-999 MET-min/wk |  |  | 1.070 |
|  | 500-749 MET-min/wk |  |  | 1.145 |
|  | 250-499 MET-min/wk |  |  | 1.225 |
|  | 0-249 MET-min/wk |  |  | 1.311 |
| Endometrium^3^ | 750-999 MET-min/wk |  | 1.120 |  |
|  | 500-749 MET-min/wk |  | 1.254 |  |
|  | 250-499 MET-min/wk |  | 1.405 |  |
|  | 0-249 MET-min/wk |  | 1.574 |  |
| Breast (post-menopausal)^3^ | 750-999 MET-min/wk |  | 1.010 |  |
|  | 500-749 MET-min/wk |  | 1.020 |  |
|  | 250-499 MET-min/wk |  | 1.030 |  |
|  | 0-249 MET-min/wk |  | 1.041 |  |
| **Vigorous-intensity physical activity^o^** |  |  |  |  |
| Breast (pre-menopausal)^3^ | 250-499 MET-min/wk |  | *1.094* |  |
|  | 0-249 MET-min/wk |  | 1.197 |  |
| **Excess body weight** | **BMI category (vs. Healthy BMI)** |  |  |  |
| Esophageal adenocarcinoma^11^  (Histology codes: 8140-8145, 8200, 8210, 8211, 8255-8323, 8480-8490, 8570-8574, 8576) | Overweight | 1.56 | 1.48 | 1.48 |
|  | Obesity | 2.43 | 2.19 | 2.19 |
| Pancreas^11^ | Overweight | 1.13 | 1.10 | 1.10 |
|  | Obesity | 1.28 | 1.21 | 1.21 |
| Liver^11^ | Overweight | 1.21 | 1.21 | 1.30 |
|  | Obesity | 1.46 | 1.46 | 1.69 |
| Colorectum^11^ | Overweight | 1.08 | 1.05 | 1.05 |
|  | Obesity | 1.17 | 1.10 | 1.10 |
| Post-menopausal breast^11^ | Overweight | - | 1.12 | - |
|  | Obesity | - | 1.25 | - |
| Uterus^11^ | Overweight | - | 1.50 | - |
|  | Obesity | - | 2.25 | - |
| Kidney^11^ | Overweight | 1.29 | 1.28 | 1.30 |
|  | Obesity | 1.66 | 1.64 | 1.69 |
| Mouth, Pharynx, Larynx^11^ | Overweight | - | - | 1.15 |
|  | Obesity | - | - | 1.32 |
| Stomach cardia^11,12^ | Overweight | 1.13 | - | 1.23 |
|  | Obesity | 1.28 | - | 1.51 |
| Gallbladder^11^ | Overweight | 1.23 | 1.25 | 1.25 |
|  | Obesity | 1.51 | 1.56 | 1.56 |
| Ovary^11^ | Overweight | - | 1.06 | - |
|  | Obesity | - | 1.12 | - |
| Advanced prostate^11^ | Overweight | 1.08 | - | - |
|  | Obesity | 1.17 | - | - |
| Thyroid^13,14^ | Overweight | 1.17 | *1.04* | 1.06 |
|  | Obesity | 1.37 | 1.08 | 1.12 |
| Multiple myeloma^15^  (Histology codes: 9732-9733) | Overweight | 1.25 | 1.14 | 1.17 |
|  | Obesity | 1.56 | 1.30 | 1.37 |
| Meningioma^16^  (Histology codes: 9530-9539) | Overweight | - | - | 1.19 |
|  | Obesity | - | - | 1.42 |

*Italics:* reported confidence interval includes null value.

Abbreviations: —, denotes that infectious agent is not causally associated with cancer site, and no relative risk was collected; MALToma, mucosa-associated lymphoid tissue lymphoma; NA, not applicable.

^a^ RR for trachea, lung, bronchus.

^b^ RR for lip and oral cavity.

^c^ RR for kidney and renal pelvis.

^d^ RR for ovary.

^e^ RR for acute myeloid leukemia.

^f^ RR for nasal-sinuses, nasopharynx.

^g^ Rg = ln(RR)/100

^h^ Rg = ln(RR)/50

^i^ Rg = ln(1/RR)/10

^j^ Rg = ln(1/RR)/200

^k^ Cervical cancer is associated with HPV-16, 18, 31, 33, 35, 39, 45, 51, 52, 56, 58, 59, 68, as opposed to the other cancer sites listed for HPV-16, which are only associated with HPV-16 infection.

^l^ Relative risk provided is for combined gastric MALToma and gastric diffuse large B cell lymphoma.

^m^ Relative risk is not applicable because the population attributable fraction is assumed to be 100%.

^n^ For moderate- or vigorous-intensity activity, the reference level was ≥1000 MET-minutes/week

^o^ For vigorous-intensity activity only, the reference level was ≥500 MET-minutes/week
