# Supplementary material for: Preventable causes of cancer in Texas by race/ethnicity: Major modifiable risk factors in the population
Source: PLoS One. 2022 Oct 13;17(10):e0274905. doi: 10.1371/journal.pone.0274905 (PMC9560474; doi:10.1371/journal.pone.0274905)
Supplement: S2 Table — (DOCX) [file pone.0274905.s009.docx]

**S2 Table.** Prevalence of alcohol consumption in Texans aged ≥18 years in 2006 (%), overall and by race/ethnicity and age group.

|  | | | **Men** | | | **Women** | | | **Persons** | | |
| --- | --- | --- | --- | --- | --- | --- | --- | --- | --- | --- | --- |
|  |  |  | **No Consumption** | **Moderate**  **Consumption** | **Heavy Consumption** | **No Consumption** | **Moderate Consumption** | **Heavy Consumption** | **No Consumption** | **Moderate Consumption** | **Heavy Consumption** |
| All | |  | 43.0 | 47.6 | 9.5 | 61.0 | 32.6 | 6.4 | 52.1 | 40.0 | 7.9 |
| Race/Ethnicity | | |  |  |  |  |  |  |  |  |  |
|  | Non-Hispanic Whites | | 37.5 | 52.4 | 10.1 | 53.9 | 39.5 | 6.6 | 45.5 | 46.1 | 8.4 |
|  | Non-Hispanic Blacks | | 54.6 | 34.8 | 10.6 | 58.1 | 38.0 | 3.9 | 56.7 | 36.8 | 6.5 |
|  | Hispanics | | 53.8 | 37.8 | 8.4 | 75.6 | 17.8 | 6.7 | 65.3 | 27.2 | 7.5 |
|  | Other Races/Ethnicities | | 47.3 | 48.1 | 4.7 | 69.0 | 26.5 | 4.5 | 56.4 | 39.0 | 4.6 |
| Age group | | |  |  |  |  |  |  |  |  |  |
|  | 18-24 years | | 47.2 | 40.2 | 12.6 | 60.8 | 30.7 | 8.6 | 53.7 | 35.7 | 10.7 |
|  | 25-34 years | | 39.1 | 51.8 | 9.1 | 55.0 | 38.3 | 6.7 | 46.8 | 45.2 | 7.9 |
|  | 35-44 years | | 40.5 | 52.5 | 7.0 | 59.9 | 33.7 | 6.4 | 50.1 | 43.2 | 6.7 |
|  | 45-54 years | | 40.8 | 49.0 | 10.3 | 54.5 | 38.0 | 7.6 | 47.5 | 43.6 | 9.0 |
|  | 55-64 years | | 40.6 | 46.9 | 12.5 | 65.4 | 30.9 | 3.8 | 53.4 | 38.7 | 8.0 |
|  | 65-74 years | | 52.1 | 40.7 | 7.2 | 70.1 | 24.1 | 5.8 | 61.8 | 31.7 | 6.4 |
|  | 75-84 years | | 56.4 | 38.7 | 4.8 | 76.5 | 20.3 | 3.2 | 69.2 | 27.0 | 3.8 |
|  | ≥85 years | | 61.1 | 38.7 | 0.2 | 79.0 | 11.2 | 9.8 | 71.1 | 23.4 | 5.5 |

Moderate consumption: more than 0 drinks/day and up to 2 drinks/day for men and 1 drink/day for women.

Heavy consumption: more than 2 drinks/day for men and 1 drink/day for women.

Note: totals may not sum manually due to rounding.
