# Supplementary material for: Preventable causes of cancer in Texas by race/ethnicity: Major modifiable risk factors in the population
Source: PLoS One. 2022 Oct 13;17(10):e0274905. doi: 10.1371/journal.pone.0274905 (PMC9560474; doi:10.1371/journal.pone.0274905)
Supplement: S3 Table — (DOCX) [file pone.0274905.s010.docx]

**S3 Table.** Prevalence of tobacco smoking in Texans aged ≥18 years in 2006 (%), overall and by race/ethnicity and age group.

|  | | **Men** | | | **Women** | | | **Persons** | | |
| --- | --- | --- | --- | --- | --- | --- | --- | --- | --- | --- |
|  |  | **Current Smoker** | **Former Smoker** | **Both** | **Current Smoker** | **Former Smoker** | **Both** | **Current Smoker** | **Former Smoker** | **Both** |
| All |  | 20.6 | 27.3 | 47.9 | 15.6 | 16.1 | 31.7 | 18.1 | 21.7 | 39.8 |
| Race/Ethnicity | |  |  |  |  |  |  |  |  |  |
|  | Non-Hispanic Whites | 17.3 | 32.5 | 49.8 | 17.8 | 20.5 | 38.3 | 17.6 | 26.7 | 44.3 |
|  | Non-Hispanic Blacks | 34.3 | 15.2 | 49.5 | 20.0 | 10.4 | 30.4 | 25.6 | 12.3 | 37.9 |
|  | Hispanics | 24.9 | 18.9 | 43.8 | 8.9 | 9.5 | 18.4 | 16.4 | 13.9 | 30.3 |
|  | Other Races/Ethnicities | 20.8 | 21.0 | 41.8 | 22.8 | 11.5 | 34.3 | 21.7 | 17.0 | 38.7 |
| Age group | |  |  |  |  |  |  |  |  |  |
|  | 18-24 years | 31.3 | 10.7 | 42.0 | 14.6 | 7.2 | 21.8 | 23.4 | 9.0 | 32.4 |
|  | 25-34 years | 18.4 | 12.2 | 30.6 | 12.8 | 9.8 | 22.6 | 15.6 | 11.0 | 26.6 |
|  | 35-44 years | 18.5 | 21.4 | 39.9 | 19.8 | 12.9 | 32.7 | 19.1 | 17.2 | 36.3 |
|  | 45-54 years | 24.1 | 32.7 | 56.8 | 20.4 | 16.1 | 36.5 | 22.3 | 24.5 | 46.8 |
|  | 55-64 years | 18.3 | 50.6 | 68.9 | 16.3 | 26.3 | 42.6 | 17.3 | 38.0 | 55.3 |
|  | 65-74 years | 11.4 | 51.1 | 62.5 | 10.5 | 26.0 | 36.5 | 10.9 | 37.5 | 48.4 |
|  | 75-84 years | 7.5 | 57.5 | 65.0 | 7.0 | 29.7 | 36.7 | 7.2 | 39.8 | 47.0 |
|  | ≥85 years | 21.2 | 51.7 | 72.9 | 6.8 | 24.7 | 31.5 | 13.2 | 36.7 | 49.9 |

Current smoker: every day or some days + smoked 100 cigarettes in lifetime.

Former smoker: smoked 100 cigarettes in lifetime + not currently smoking.
