# Supplementary material for: Preventable causes of cancer in Texas by race/ethnicity: Major modifiable risk factors in the population
Source: PLoS One. 2022 Oct 13;17(10):e0274905. doi: 10.1371/journal.pone.0274905 (PMC9560474; doi:10.1371/journal.pone.0274905)
Supplement: S4 Table — (DOCX) [file pone.0274905.s011.docx]

**S4 Table**. Prevalence of Americans aged ≥18 years not meeting recommendations for red meat, processed meat, fiber, and calcium consumption (%), overall and by race/ethnicity.

|  | | **Men** | | | | **Women** | | | | **Persons** | | | |
| --- | --- | --- | --- | --- | --- | --- | --- | --- | --- | --- | --- | --- | --- |
|  |  | **Red Meat^a^** | **Processed Meat^a^** | **Fiber** | **Calcium** | **Red Meat^a^** | **Processed Meat^a^** | **Fiber** | **Calcium** | **Red Meat^a^** | **Processed Meat^a^** | **Fiber** | **Calcium** |
| All |  | 59.9 | 88.1 | 88.0 | 53.4 | 45.4 | 83.9 | 95.6 | 71.7 | 52.7 | 86.0 | 92.0 | 63.0 |
| Race/Ethnicity | |  |  |  |  |  |  |  |  |  |  |  |  |
|  | Non-Hispanic Whites | 63.9 | 91.6 | 87.8 | 49.5 | 48.7 | 85.9 | 95.6 | 69.3 | 56.4 | 88.8 | 91.9 | 59.9 |
|  | Non-Hispanic Blacks | 49.0 | 86.6 | 96.4 | 67.4 | 37.6 | 86.7 | 98.3 | 86.6 | 42.9 | 86.6 | 97.5 | 78.2 |
|  | Hispanics | 52.8 | 84.9 | 79.9 | 63.6 | 40.8 | 79.8 | 92.9 | 69.0 | 47.3 | 82.5 | 86.6 | 66.4 |
|  | Other Races/Ethnicities | 53.8 | 62.5 | 89.3 | 57.2 | 36.6 | 67.9 | 94.0 | 74.6 | 44.9 | 65.3 | 91.7 | 66.2 |

Red meat recommended consumption: ≤60 grams/day.

Processed meat recommended consumption: 0 grams/day.

Fiber recommended intake: ≥28 grams/day.

Calcium recommended intake: ≥1000 milligrams/day.

^a^ Prevalence data sourced from NHANES 2009-2010 given evidence that consumption has remained stable since 2006. Prevalence data only available for adults aged 18-69.

Note: totals may not sum manually due to rounding.
