# Supplementary material for: Preventable causes of cancer in Texas by race/ethnicity: Major modifiable risk factors in the population
Source: PLoS One. 2022 Oct 13;17(10):e0274905. doi: 10.1371/journal.pone.0274905 (PMC9560474; doi:10.1371/journal.pone.0274905)
Supplement: S5 Table — (DOCX) [file pone.0274905.s012.docx]

**S5 Table.** Prevalence (shown as percentage, %) of oncogenic infections by race/ethnicity in the U.S. in 2006 according to the National Health and Nutrition Examination Survey, in men, women, and persons aged ≥18 years.

| **Race/Ethnicity** | **Men** | | | | **Women** | | | | **Persons** | | | |
| --- | --- | --- | --- | --- | --- | --- | --- | --- | --- | --- | --- | --- |
|  | **HPV-16**  (L1) | ***H. pylori*** (IgG)^a^ | **HCV** (RNA + anti-HCV) | **HBV**  (HBsAg) | **HPV-16**  (L1) | ***H. pylori*** (IgG)^a^ | **HCV** (RNA + anti-HCV) | **HBV**  (HBsAg) | **HPV-16**  (L1) | ***H. pylori*** (IgG)^a^ | **HCV** (RNA + anti-HCV) | **HBV**  (HBsAg) |
| All | 5.2 | 30.6 | 1.6 | 0.5 | 15.3 | 29.8 | 0.6 | 0.2 | 10.3 | 30.1 | 1.1 | 0.4 |
| Non-Hispanic Whites | 5.4 | 19.7 | 1.6 | 0.4 | 15.3 | 21.5 | 0.2 | 0.1 | 10.4 | 20.6 | 0.9 | 0.2 |
| Non-Hispanic Blacks | 6.2 | 55.9 | 2.6 | 1.1 | 23.3 | 48.4 | 2.5 | 0.9 | 15.4 | 51.9 | 2.5 | 1.0 |
| Hispanics | 3.6 | 62.6 | 1.0 | 0.2 | 10.1 | 53.0 | 0.2 | 0.0 | 6.8 | 57.5 | 0.6 | 0.1 |
| Other Races/Ethnicities | 5.2 | 45.8 | 0.0 | 3.1 | 8.9 | 38.0 | 2.1 | 0.1 | 7.3 | 41.9 | 1.2 | 1.4 |

Abbreviations: HPV-16, human papillomavirus strain 16; *H. pylori,* *Helicobacter pylori*; IgG, immunoglobulin G; HCV, hepatitis C virus; RNA, ribonucleic acid; HBV, hepatitis B virus; HBsAg, hepatitis B virus surface antigen.

^a^ Prevalence data were sourced from NHANES 1999-2000 given evidence that prevalence has remained stable through 2006.
