# Supplementary material for: Preventable causes of cancer in Texas by race/ethnicity: Major modifiable risk factors in the population
Source: PLoS One. 2022 Oct 13;17(10):e0274905. doi: 10.1371/journal.pone.0274905 (PMC9560474; doi:10.1371/journal.pone.0274905)
Supplement: S6 Table — (DOCX) [file pone.0274905.s013.docx]

**S6 Table.** Prevalence of moderate- or vigorous-intensity physical activity in Texans aged ≥18 years in 2005 (%), overall and by race/ethnicity and age group.

| **MET-min/wk** | | **Men** | | | | | **Women** | | | | | **Persons** | | | | |
| --- | --- | --- | --- | --- | --- | --- | --- | --- | --- | --- | --- | --- | --- | --- | --- | --- |
|  |  | **≥1000** | **750-999** | **500-749** | **250-499** | **0-249** | **≥1000** | **750-999** | **500-749** | **250-499** | **0-249** | **≥1000** | **750-999** | **500-749** | **250-499** | **0-249** |
| All |  | 47.2 | 4.1 | 13.0 | 12.1 | 23.7 | 36.4 | 3.6 | 14.7 | 16.7 | 28.6 | 41.7 | 3.9 | 13.8 | 14.4 | 26.2 |
| Race/Ethnicity | |  |  |  |  |  |  |  |  |  |  |  |  |  |  |  |
|  | Non-Hispanic Whites | 48.5 | 4.3 | 13.9 | 12.5 | 20.8 | 36.6 | 4.6 | 15.3 | 17.5 | 26.0 | 42.4 | 4.4 | 14.6 | 15.1 | 23.5 |
|  | Non-Hispanic Blacks | 41.8 | 5.0 | 12.9 | 12.9 | 27.5 | 35.3 | 2.6 | 11.4 | 12.5 | 38.3 | 38.2 | 3.7 | 12.1 | 12.7 | 33.4 |
|  | Hispanics | 46.3 | 3.5 | 11.8 | 10.5 | 27.9 | 36.6 | 2.1 | 13.5 | 16.2 | 31.6 | 41.4 | 2.8 | 12.7 | 13.4 | 29.7 |
|  | Other Races/Ethnicities | 38.6 | 5.7 | 10.9 | 19.2 | 25.7 | 32.7 | 4.6 | 22.3 | 17.3 | 23.2 | 36.0 | 5.2 | 15.9 | 18.4 | 24.6 |
| Age group | |  |  |  |  |  |  |  |  |  |  |  |  |  |  |  |
|  | 18-24 years | 60.0 | 1.6 | 8.7 | 9.5 | 20.2 | 38.1 | 3.4 | 15.8 | 19.7 | 23.0 | 49.4 | 2.5 | 12.1 | 14.5 | 21.6 |
|  | 25-34 years | 45.6 | 5.8 | 15.1 | 14.2 | 19.4 | 41.4 | 3.2 | 15.5 | 18.0 | 21.9 | 43.5 | 4.5 | 15.3 | 16.1 | 20.6 |
|  | 35-44 years | 49.8 | 5.5 | 13.7 | 12.7 | 18.4 | 38.5 | 4.4 | 16.4 | 16.2 | 24.5 | 44.2 | 4.9 | 15.1 | 14.5 | 21.4 |
|  | 45-54 years | 43.2 | 3.5 | 11.8 | 12.6 | 29.0 | 38.4 | 4.8 | 12.1 | 16.6 | 28.0 | 40.8 | 4.2 | 11.9 | 14.6 | 28.5 |
|  | 55-64 years | 40.8 | 4.0 | 14.6 | 10.6 | 30.1 | 31.6 | 3.4 | 16.2 | 16.0 | 32.8 | 36.1 | 3.7 | 15.4 | 13.4 | 31.5 |
|  | 65-74 years | 46.3 | 2.8 | 14.1 | 11.4 | 25.4 | 27.8 | 3.4 | 11.7 | 14.5 | 42.5 | 36.4 | 3.1 | 12.8 | 13.1 | 34.7 |
|  | 75-84 years | 36.3 | 4.3 | 11.1 | 11.8 | 36.5 | 28.8 | 0.4 | 13.6 | 11.1 | 46.1 | 31.7 | 1.9 | 12.6 | 11.4 | 42.4 |
|  | ≥85 years | 25.5 | 0.0 | 21.8 | 13.2 | 39.5 | 20.6 | 5.9 | 8.4 | 19.1 | 46.1 | 22.4 | 3.8 | 13.3 | 16.9 | 43.7 |

Note: totals may not sum manually due to rounding.
