# Supplementary material for: Preventable causes of cancer in Texas by race/ethnicity: Major modifiable risk factors in the population
Source: PLoS One. 2022 Oct 13;17(10):e0274905. doi: 10.1371/journal.pone.0274905 (PMC9560474; doi:10.1371/journal.pone.0274905)
Supplement: S7 Table — (DOCX) [file pone.0274905.s014.docx]

**S7 Table.** Prevalence of overweight and obesity among adults aged ≥18 years in Texas in 2006 (%), overall and by race/ethnicity and age group.

|  | **Men** | | | **Women** | | | **Persons** | | |
| --- | --- | --- | --- | --- | --- | --- | --- | --- | --- |
| **Race/Ethnicity** | **Overweight** | **Obese** | **Both** | **Overweight** | **Obese** | **Both** | **Overweight** | **Obese** | **Both** |
| All | 42.1 | 26.6 | 68.7 | 30.2 | 25.7 | 55.9 | 36.2 | 26.2 | 62.4 |
|  |  |  |  |  |  |  |  |  |  |
| Non-Hispanic Whites | 45.5 | 23.5 | 69.0 | 28.8 | 22.1 | 50.9 | 37.5 | 22.8 | 60.3 |
| Non-Hispanic Blacks | 35.2 | 40.4 | 75.6 | 30.0 | 41.2 | 71.2 | 32.2 | 40.9 | 73.1 |
| Hispanics | 37.5 | 29.4 | 66.9 | 34.8 | 29.4 | 64.2 | 36.1 | 29.4 | 65.5 |
| Other Races/Ethnicities | 25.9 | 34.8 | 60.7 | 18.5 | 21.9 | 40.4 | 22.6 | 29.0 | 51.6 |
|  |  |  |  |  |  |  |  |  |  |
| 18-24 years | 31.0 | 15.8 | 46.8 | 20.4 | 18.2 | 38.6 | 26.0 | 16.9 | 42.9 |
| 25-34 years | 43.9 | 24.3 | 68.2 | 25.7 | 24.9 | 50.6 | 35.3 | 24.5 | 59.8 |
| 35-44 years | 47.6 | 31.6 | 79.2 | 33.0 | 28.7 | 61.7 | 40.5 | 30.2 | 70.7 |
| 45-54 years | 42.1 | 30.8 | 72.9 | 30.7 | 29.8 | 60.5 | 36.7 | 30.4 | 67.1 |
| 55-64 years | 39.1 | 32.9 | 72.0 | 34.8 | 31.1 | 65.9 | 36.9 | 32.0 | 68.9 |
| 65-74 years | 51.7 | 25.6 | 77.3 | 36.1 | 27.8 | 63.9 | 43.5 | 26.8 | 70.3 |
| 75-84 years | 40.2 | 21.5 | 61.7 | 36.7 | 15.1 | 51.8 | 38.0 | 17.5 | 55.5 |
| ≥85 years | 39.1 | 6.3 | 45.4 | 34.7 | 7.4 | 42.1 | 36.7 | 6.9 | 43.6 |

Overweight: body mass index 25.0-29.9; Obese: body mass index ≥30.0.
