# Supplementary material for: Preventable causes of cancer in Texas by race/ethnicity: Major modifiable risk factors in the population
Source: PLoS One. 2022 Oct 13;17(10):e0274905. doi: 10.1371/journal.pone.0274905 (PMC9560474; doi:10.1371/journal.pone.0274905)
Supplement: S8 Table — (DOCX) [file pone.0274905.s015.docx]

**S8 Table.** Age-weighted PAFs of cancers attributable to modifiable risk factors in Texas in 2015 for all races/ethnicities (%), adults aged ≥25 years.

| **All Races/Ethnicities** | **Lung, Bronchus** | **Mouth, Pharynx, Larynx** | **Esophagus** | **Stomach** | **Pancreas** | **Colorectum** | **Liver** | **Kidney, Renal Pelvis, Ureter** | **Bladder** | **Ovary** | **Myeloid Leukemia** | **Nasal Cavity, Accessory Sinuses** | **Breast** | **Uterus** | **Gallbladder** | **Prostate** | **Thyroid** | **Multiple Myeloma** | **Meningioma** | **Vulva** | **Vagina** | **Penis** | **Anus** | **Cervix** | **NHL** | **Kaposi Sarcoma** | **All Cancers*** |
| --- | --- | --- | --- | --- | --- | --- | --- | --- | --- | --- | --- | --- | --- | --- | --- | --- | --- | --- | --- | --- | --- | --- | --- | --- | --- | --- | --- |
| **Men** | | | | | | | | | | | | | | | | | | | | | | | | | | | |
| **Tobacco Smoking** | 86.9 | 58.2 | 54.2 | 26.5 | 9.3 | 13.1 | 30.8 | 25.3 | 52.5 | - | 23.9 | 24.3 | - | - |  |  |  |  |  | - | - |  |  | - |  |  | **23.3** |
| **Overweight & Obesity** |  | 11.6 | 22.9 | 3.9 | 12.1 | 7.7 | 18.9 | 23.9 |  | - |  |  | - | - | 20.0 | 2.6 | 15.0 | 21.5 | 17.4 | - | - |  |  | - |  |  | **5.8** |
| **Alcohol Consumption** |  | 16.2 | 11.6 | 4.2 |  | 12.9 | 4.3 |  |  | - |  |  | - | - |  |  |  |  |  | - | - |  |  | - |  |  | **2.8** |
| **Insufficient Physical Activity** |  |  |  |  |  | 7.7 |  |  |  | - |  |  | - | - |  |  |  |  |  | - | - |  |  | - |  |  | **0.8** |
| **HPV Infection** |  | 7.9 |  |  |  |  |  |  |  | - |  |  | - | - |  |  |  |  |  | - | - | 31.0 | 17.3 | - |  |  | **0.6** |
| **Insufficient Fiber Intake** |  |  |  |  |  | 12.3 |  |  |  | - |  |  | - | - |  |  |  |  |  | - | - |  |  | - |  |  | **1.3** |
| **Processed Meat Consumption** |  |  |  |  |  | 9.0 |  |  |  | - |  |  | - | - |  |  |  |  |  | - | - |  |  | - |  |  | **1.0** |
| **Chronic HCV Infection** |  |  |  |  |  |  | 32.5 |  |  | - |  |  | - | - |  |  |  |  |  | - | - |  |  | - | 1.2 |  | **1.4** |
| **Insufficient Calcium Intake** |  |  |  |  |  | 6.6 |  |  |  | - |  |  | - | - |  |  |  |  |  | - | - |  |  | - |  |  | **0.7** |
| **Chronic H. pylori Infection** |  |  |  | 25.6 |  |  |  |  |  | - |  |  | - | - |  |  |  |  |  | - | - |  |  | - | 1.1 |  | **0.6** |
| **Red Meat Consumption** |  |  |  |  |  | 6.5 |  |  |  | - |  |  | - | - |  |  |  |  |  | - | - |  |  | - |  |  | **0.7** |
| **Chronic HBV Infection** |  |  |  |  |  |  | 14.0 |  |  | - |  |  | - | - |  |  |  |  |  | - | - |  |  | - |  |  | **0.6** |
| **HHV-8 Infection** |  |  |  |  |  |  |  |  |  | - |  |  | - | - |  |  |  |  |  | - | - |  |  | - |  | 100.0 | **0.2** |
| **All Factors** | **86.9** | **71.5** | **68.7** | **49.6** | **20.3** | **55.1** | **68.8** | **43.1** | **52.5** | **-** | **23.9** | **24.3** | **-** | **-** | **20.0** | **2.6** | **15.0** | **21.5** | **17.4** | **-** | **-** | **31.0** | **17.3** | **-** | **2.3** | **100.0** | **35.1** |
| **Women** | | | | | | | | | | | | | | | | | | | | | | | | | | | |
| **Tobacco Smoking** | 81.9 | 56.2 | 46.5 | 11.3 | 15.0 | 11.9 | 12.8 | 6.8 | 41.3 | 0.2 | 3.4 | 18.7 |  |  |  | - |  |  |  |  |  | - |  | 17.8 |  |  | **13.5** |
| **Overweight & Obesity** |  | 11.1 | 10.0 | 2.5 | 8.4 | 4.3 | 16.7 | 20.8 |  | 5.1 |  |  | 9.0 | 34.1 | 19.6 | - | 3.2 | 11.6 | 15.0 |  |  | - |  |  |  |  | **7.4** |
| **Alcohol Consumption** |  | 25.9 | 14.8 | 1.2 |  | 2.5 | 19.9 |  |  |  |  |  | 6.2 |  |  | - |  |  |  |  |  | - |  |  |  |  | **3.0** |
| **Insufficient Physical Activity** |  |  |  |  |  | 10.3 |  |  |  |  |  |  | 3.1 | 21.7 |  | - |  |  |  |  |  | - |  |  |  |  | **3.2** |
| **HPV Infection** |  | 12.2 |  |  |  |  |  |  |  |  |  |  |  |  |  | - |  |  |  | 22.7 | 34.7 | - | 34.7 | 100.0 |  |  | **2.9** |
| **Insufficient Fiber Intake** |  |  |  |  |  | 12.1 |  |  |  |  |  |  |  |  |  | - |  |  |  |  |  | - |  |  |  |  | **1.0** |
| **Processed Meat Consumption** |  |  |  |  |  | 11.1 |  |  |  |  |  |  |  |  |  | - |  |  |  |  |  | - |  |  |  |  | **1.0** |
| **Chronic HCV Infection** |  |  |  |  |  |  | 10.9 |  |  |  |  |  |  |  |  | - |  |  |  |  |  | - |  |  | 0.4 |  | **0.2** |
| **Insufficient Calcium Intake** |  |  |  |  |  | 9.9 |  |  |  |  |  |  |  |  |  | - |  |  |  |  |  | - |  |  |  |  | **0.8** |
| **Chronic H. pylori Infection** |  |  |  | 31.4 |  |  |  |  |  |  |  |  |  |  |  | - |  |  |  |  |  | - |  |  | 1.1 |  | **0.5** |
| **Red Meat Consumption** |  |  |  |  |  | 0.3 |  |  |  |  |  |  |  |  |  | - |  |  |  |  |  | - |  |  |  |  | **0.0** |
| **Chronic HBV Infection** |  |  |  |  |  |  | 3.2 |  |  |  |  |  |  |  |  | - |  |  |  |  |  | - |  |  |  |  | **0.0** |
| **HHV-8 Infection** |  |  |  |  |  |  |  |  |  |  |  |  |  |  |  | - |  |  |  |  |  | - |  |  |  | 100.0 | **0.0** |
| **All Factors** | **81.9** | **74.7** | **59.0** | **41.3** | **22.2** | **48.3** | **49.8** | **26.2** | **41.3** | **5.2** | **3.4** | **18.7** | **17.2** | **48.4** | **19.6** | **-** | **3.2** | **11.6** | **15.0** | **22.7** | **34.7** | **-** | **34.7** | **100.0** | **1.5** | **100.0** | **29.5** |
| **Persons** | | | | | | | | | | | | | | | | | | | | | | | | | | | |
| **Tobacco Smoking** | 84.7 | 57.7 | 52.6 | 20.2 | 12.1 | 12.6 | 26.1 | 18.2 | 49.8 | 0.2 | 15.1 | 22.5 |  |  |  |  |  |  |  |  |  |  |  | 17.8 |  |  | **18.4** |
| **Overweight & Obesity** |  | 11.5 | 20.2 | 3.3 | 10.3 | 6.2 | 18.3 | 22.7 |  | 5.1 |  |  | 9.0 | 34.1 | 19.7 | 2.6 | 6.2 | 16.9 | 16.0 |  |  |  |  |  |  |  | **6.6** |
| **Alcohol Consumption** |  | 18.6 | 12.3 | 2.9 |  | 8.3 | 8.4 |  |  |  |  |  | 6.2 |  |  |  |  |  |  |  |  |  |  |  |  |  | **2.9** |
| **Insufficient Physical Activity** |  |  |  |  |  | 8.8 |  |  |  |  |  |  | 3.1 | 21.7 |  |  |  |  |  |  |  |  |  |  |  |  | **2.0** |
| **HPV Infection** |  | 9.0 |  |  |  |  |  |  |  |  |  |  |  |  |  |  |  |  |  | 22.7 | 34.7 | 31.0 | 27.3 | 100.0 |  |  | **1.8** |
| **Insufficient Fiber Intake** |  |  |  |  |  | 12.3 |  |  |  |  |  |  |  |  |  |  |  |  |  |  |  |  |  |  |  |  | **1.2** |
| **Processed Meat Consumption** |  |  |  |  |  | 9.9 |  |  |  |  |  |  |  |  |  |  |  |  |  |  |  |  |  |  |  |  | **1.0** |
| **Chronic HCV Infection** |  |  |  |  |  |  | 26.9 |  |  |  |  |  |  |  |  |  |  |  |  |  |  |  |  |  | 0.9 |  | **0.8** |
| **Insufficient Calcium Intake** |  |  |  |  |  | 8.0 |  |  |  |  |  |  |  |  |  |  |  |  |  |  |  |  |  |  |  |  | **0.8** |
| **Chronic H. pylori Infection** |  |  |  | 28.0 |  |  |  |  |  |  |  |  |  |  |  |  |  |  |  |  |  |  |  |  | 1.1 |  | **0.5** |
| **Red Meat Consumption** |  |  |  |  |  | 3.8 |  |  |  |  |  |  |  |  |  |  |  |  |  |  |  |  |  |  |  |  | **0.4** |
| **Chronic HBV Infection** |  |  |  |  |  |  | 11.1 |  |  |  |  |  |  |  |  |  |  |  |  |  |  |  |  |  |  |  | **0.3** |
| **HHV-8 Infection** |  |  |  |  |  |  |  |  |  |  |  |  |  |  |  |  |  |  |  |  |  |  |  |  |  | 100.0 | **0.1** |
| **All Factors** | **84.7** | **72.3** | **66.8** | **46.0** | **21.1** | **52.0** | **64.0** | **36.8** | **49.8** | **5.2** | **15.1** | **22.5** | **17.2** | **48.4** | **19.7** | **2.6** | **6.2** | **16.9** | **16.0** | **22.7** | **34.7** | **31.0** | **27.3** | **100.0** | **2.0** | **100.0** | **32.3** |

*Excluding basal cell carcinoma and squamous cell carcinoma of the skin. All cancers combined are displayed as PAF (excess cases).
