# Supplementary material for: Preventable causes of cancer in Texas by race/ethnicity: Major modifiable risk factors in the population
Source: PLoS One. 2022 Oct 13;17(10):e0274905. doi: 10.1371/journal.pone.0274905 (PMC9560474; doi:10.1371/journal.pone.0274905)
Supplement: S9 Table — (DOCX) [file pone.0274905.s016.docx]

**S9 Table.** Age-weighted PAFs of cancers attributable to modifiable risk factors in Texas in 2015 for non-Hispanic Whites (%), adults aged ≥25 years.

| **Non-Hispanic Whites** | **Lung, Bronchus** | **Mouth, Pharynx, Larynx** | **Esophagus** | **Stomach** | **Pancreas** | **Colorectum** | **Liver** | **Kidney, Renal Pelvis, Ureter** | **Bladder** | **Ovary** | **Myeloid Leukemia** | **Nasal Cavity, Accessory Sinuses** | **Breast** | **Uterus** | **Gallbladder** | **Prostate** | **Thyroid** | **Multiple Myeloma** | **Meningioma** | **Vulva** | **Vagina** | **Penis** | **Anus** | **Cervix** | **NHL** | **Kaposi Sarcoma** | **All Cancers*** |
| --- | --- | --- | --- | --- | --- | --- | --- | --- | --- | --- | --- | --- | --- | --- | --- | --- | --- | --- | --- | --- | --- | --- | --- | --- | --- | --- | --- |
| **Men** | | | | | | | | | | | | | | | | | | | | | | | | | | | |
| **Tobacco Smoking** | 86.5 | 55.5 | 53.8 | 26.6 | 8.1 | 12.9 | 29.6 | 25.2 | 52.4 | - | 23.8 | 24.3 | - | - |  |  |  |  |  | - | - |  |  | - |  |  | **24.4** |
| **Overweight & Obesity** |  | 11.0 | 24.2 | 5.8 | 11.6 | 7.4 | 18.3 | 22.9 |  | - |  |  | - | - | 19.3 | 2.5 | 14.5 | 20.7 | 16.6 | - | - |  |  | - |  |  | **5.2** |
| **Alcohol Consumption** |  | 16.7 | 8.7 | 4.4 |  | 13.3 | 4.7 |  |  | - |  |  | - | - |  |  |  |  |  | - | - |  |  | - |  |  | **2.7** |
| **Insufficient Physical Activity** |  |  |  |  |  | 7.5 |  |  |  | - |  |  | - | - |  |  |  |  |  | - | - |  |  | - |  |  | **0.7** |
| **HPV Infection** |  | 8.3 |  |  |  |  |  |  |  | - |  |  | - | - |  |  |  |  |  | - | - | 29.0 | 16.6 | - |  |  | **0.6** |
| **Insufficient Fiber Intake** |  |  |  |  |  | 12.1 |  |  |  | - |  |  | - | - |  |  |  |  |  | - | - |  |  | - |  |  | **1.2** |
| **Processed Meat Consumption** |  |  |  |  |  | 9.4 |  |  |  | - |  |  | - | - |  |  |  |  |  | - | - |  |  | - |  |  | **0.9** |
| **Chronic HCV Infection** |  |  |  |  |  |  | 27.4 |  |  | - |  |  | - | - |  |  |  |  |  | - | - |  |  | - | 0.9 |  | **0.8** |
| **Insufficient Calcium Intake** |  |  |  |  |  | 5.8 |  |  |  | - |  |  | - | - |  |  |  |  |  | - | - |  |  | - |  |  | **0.6** |
| **Chronic H. pylori Infection** |  |  |  | 15.3 |  |  |  |  |  | - |  |  | - | - |  |  |  |  |  | - | - |  |  | - | 0.8 |  | **0.3** |
| **Red Meat Consumption** |  |  |  |  |  | 6.8 |  |  |  | - |  |  | - | - |  |  |  |  |  | - | - |  |  | - |  |  | **0.7** |
| **Chronic HBV Infection** |  |  |  |  |  |  | 10.5 |  |  | - |  |  | - | - |  |  |  |  |  | - | - |  |  | - |  |  | **0.3** |
| **HHV-8 Infection** |  |  |  |  |  |  |  |  |  | - |  |  | - | - |  |  |  |  |  | - | - |  |  | - |  | 100.0 | **0.1** |
| **All Factors** | **86.5** | **69.7** | **68.0** | **44.0** | **18.8** | **54.8** | **64.4** | **42.4** | **52.4** | **-** | **23.8** | **24.3** | **-** | **-** | **19.3** | **2.5** | **14.5** | **20.7** | **16.6** | **-** | **-** | **29.0** | **16.6** | **-** | **1.8** | **100.0** | **34.5** |
| **Women** | | | | | | | | | | | | | | | | | | | | | | | | | | | |
| **Tobacco Smoking** | 82.6 | 58.7 | 48.1 | 11.7 | 15.7 | 12.6 | 13.4 | 7.5 | 42.9 | 0.2 | 3.9 | 19.8 |  |  |  | - |  |  |  |  |  | - |  | 20.8 |  |  | **15.8** |
| **Overweight & Obesity** |  | 9.9 | 10.2 | 3.2 | 7.6 | 3.9 | 15.1 | 18.8 |  | 4.6 |  |  | 8.3 | 31.6 | 17.8 | - | 2.8 | 10.5 | 13.2 |  |  | - |  |  |  |  | **6.3** |
| **Alcohol Consumption** |  | 22.7 | 11.5 | 1.0 |  | 2.3 | 17.7 |  |  |  |  |  | 5.9 |  |  | - |  |  |  |  |  | - |  |  |  |  | **2.7** |
| **Insufficient Physical Activity** |  |  |  |  |  | 10.0 |  |  |  |  |  |  | 2.7 | 21.1 |  | - |  |  |  |  |  | - |  |  |  |  | **2.8** |
| **HPV Infection** |  | 12.0 |  |  |  |  |  |  |  |  |  |  |  |  |  | - |  |  |  | 21.6 | 32.1 | - | 33.3 | 100.0 |  |  | **2.2** |
| **Insufficient Fiber Intake** |  |  |  |  |  | 12.0 |  |  |  |  |  |  |  |  |  | - |  |  |  |  |  | - |  |  |  |  | **1.0** |
| **Processed Meat Consumption** |  |  |  |  |  | 11.3 |  |  |  |  |  |  |  |  |  | - |  |  |  |  |  | - |  |  |  |  | **0.9** |
| **Chronic HCV Infection** |  |  |  |  |  |  | 1.6 |  |  |  |  |  |  |  |  | - |  |  |  |  |  | - |  |  | 0.1 |  | **0.0** |
| **Insufficient Calcium Intake** |  |  |  |  |  | 9.3 |  |  |  |  |  |  |  |  |  | - |  |  |  |  |  | - |  |  |  |  | **0.8** |
| **Chronic H. pylori Infection** |  |  |  | 25.3 |  |  |  |  |  |  |  |  |  |  |  | - |  |  |  |  |  | - |  |  | 1.1 |  | **0.3** |
| **Red Meat Consumption** |  |  |  |  |  | 0.4 |  |  |  |  |  |  |  |  |  | - |  |  |  |  |  | - |  |  |  |  | **0.0** |
| **Chronic HBV Infection** |  |  |  |  |  |  | 0.1 |  |  |  |  |  |  |  |  | - |  |  |  |  |  | - |  |  |  |  | **0.0** |
| **HHV-8 Infection** |  |  |  |  |  |  |  |  |  |  |  |  |  |  |  | - |  |  |  |  |  | - |  |  |  | 100.0 | **0.0** |
| **All Factors** | **82.6** | **74.7** | **58.9** | **36.8** | **22.1** | **48.0** | **40.4** | **24.8** | **42.9** | **4.8** | **3.9** | **19.8** | **16.1** | **46.0** | **17.8** | **-** | **2.8** | **10.5** | **13.2** | **21.6** | **32.1** | **-** | **33.3** | **100.0** | **1.2** | **100.0** | **29.2** |
| **Persons** | | | | | | | | | | | | | | | | | | | | | | | | | | | |
| **Tobacco Smoking** | 84.7 | 56.2 | 52.7 | 21.0 | 11.7 | 12.8 | 25.4 | 18.8 | 50.2 | 0.2 | 15.4 | 22.6 |  |  |  |  |  |  |  |  |  |  |  | 20.8 |  |  | **20.2** |
| **Overweight & Obesity** |  | 10.7 | 21.4 | 4.8 | 9.7 | 5.8 | 17.5 | 21.4 |  | 4.6 |  |  | 8.3 | 31.6 | 18.4 | 2.5 | 6.2 | 16.0 | 14.9 |  |  |  |  |  |  |  | **5.8** |
| **Alcohol Consumption** |  | 18.2 | 9.3 | 3.1 |  | 8.4 | 8.1 |  |  |  |  |  | 5.9 |  |  |  |  |  |  |  |  |  |  |  |  |  | **2.7** |
| **Insufficient Physical Activity** |  |  |  |  |  | 8.6 |  |  |  |  |  |  | 2.7 | 21.1 |  |  |  |  |  |  |  |  |  |  |  |  | **1.8** |
| **HPV Infection** |  | 9.2 |  |  |  |  |  |  |  |  |  |  |  |  |  |  |  |  |  | 21.6 | 32.1 | 29.0 | 26.7 | 100.0 |  |  | **1.4** |
| **Insufficient Fiber Intake** |  |  |  |  |  | 12.0 |  |  |  |  |  |  |  |  |  |  |  |  |  |  |  |  |  |  |  |  | **1.1** |
| **Processed Meat Consumption** |  |  |  |  |  | 10.3 |  |  |  |  |  |  |  |  |  |  |  |  |  |  |  |  |  |  |  |  | **0.9** |
| **Chronic HCV Infection** |  |  |  |  |  |  | 20.7 |  |  |  |  |  |  |  |  |  |  |  |  |  |  |  |  |  | 0.6 |  | **0.4** |
| **Insufficient Calcium Intake** |  |  |  |  |  | 7.4 |  |  |  |  |  |  |  |  |  |  |  |  |  |  |  |  |  |  |  |  | **0.7** |
| **Chronic H. pylori Infection** |  |  |  | 19.0 |  |  |  |  |  |  |  |  |  |  |  |  |  |  |  |  |  |  |  |  | 0.9 |  | **0.3** |
| **Red Meat Consumption** |  |  |  |  |  | 3.9 |  |  |  |  |  |  |  |  |  |  |  |  |  |  |  |  |  |  |  |  | **0.4** |
| **Chronic HBV Infection** |  |  |  |  |  |  | 7.8 |  |  |  |  |  |  |  |  |  |  |  |  |  |  |  |  |  |  |  | **0.2** |
| **HHV-8 Infection** |  |  |  |  |  |  |  |  |  |  |  |  |  |  |  |  |  |  |  |  |  |  |  |  |  | 100.0 | **0.0** |
| **All Factors** | **84.7** | **71.0** | **66.3** | **41.0** | **20.2** | **51.7** | **58.6** | **36.2** | **50.2** | **4.8** | **15.4** | **22.6** | **16.1** | **46.0** | **18.4** | **2.5** | **6.2** | **16.0** | **14.9** | **21.6** | **32.1** | **29.0** | **26.7** | **100.0** | **1.5** | **100.0** | **31.9** |

*Excluding basal cell carcinoma and squamous cell carcinoma of the skin. All cancers combined are displayed as PAF (excess cases).
