# Supplementary material for: Preventable causes of cancer in Texas by race/ethnicity: Major modifiable risk factors in the population
Source: PLoS One. 2022 Oct 13;17(10):e0274905. doi: 10.1371/journal.pone.0274905 (PMC9560474; doi:10.1371/journal.pone.0274905)
Supplement: S10 Table — (DOCX) [file pone.0274905.s017.docx]

**S10 Table.** Age-weighted PAFs of cancers attributable to modifiable risk factors in Texas in 2015 for non-Hispanic Blacks (%), adults aged ≥25 years.

| **Non-Hispanic Blacks** | **Lung, Bronchus** | **Mouth, Pharynx, Larynx** | **Esophagus** | **Stomach** | **Pancreas** | **Colorectum** | **Liver** | **Kidney, Renal Pelvis, Ureter** | **Bladder** | **Ovary** | **Myeloid Leukemia** | **Nasal Cavity, Accessory Sinuses** | **Breast** | **Uterus** | **Gallbladder** | **Prostate** | **Thyroid** | **Multiple Myeloma** | **Meningioma** | **Vulva** | **Vagina** | **Penis** | **Anus** | **Cervix** | **NHL** | **Kaposi Sarcoma** | **All Cancers*** |
| --- | --- | --- | --- | --- | --- | --- | --- | --- | --- | --- | --- | --- | --- | --- | --- | --- | --- | --- | --- | --- | --- | --- | --- | --- | --- | --- | --- |
| **Men** | | | | | | | | | | | | | | | | | | | | | | | | | | | |
| **Tobacco Smoking** | 87.5 | 67.6 | 53.2 | 28.1 | 16.9 | 15.0 | 39.0 | 27.3 | 51.0 | - | 27.3 | 28.0 | - | - |  |  |  |  |  | - | - |  |  | - |  |  | **24.4** |
| **Overweight & Obesity** |  | 14.1 | 9.0 | 2.3 | 13.6 | 8.9 | 21.4 | 27.2 |  | - |  |  | - | - | 22.0 | 3.1 | 17.7 | 23.5 | 20.2 | - | - |  |  | - |  |  | **6.7** |
| **Alcohol Consumption** |  | 13.5 | 21.9 | 4.6 |  | 12.2 | 2.9 |  |  | - |  |  | - | - |  |  |  |  |  | - | - |  |  | - |  |  | **2.5** |
| **Insufficient Physical Activity** |  |  |  |  |  | 9.2 |  |  |  | - |  |  | - | - |  |  |  |  |  | - | - |  |  | - |  |  | **1.1** |
| **HPV Infection** |  | 7.5 |  |  |  |  |  |  |  | - |  |  | - | - |  |  |  |  |  | - | - | 44.1 | 22.4 | - |  |  | **0.5** |
| **Insufficient Fiber Intake** |  |  |  |  |  | 14.9 |  |  |  | - |  |  | - | - |  |  |  |  |  | - | - |  |  | - |  |  | **1.8** |
| **Processed Meat Consumption** |  |  |  |  |  | 9.5 |  |  |  | - |  |  | - | - |  |  |  |  |  | - | - |  |  | - |  |  | **1.1** |
| **Chronic HCV Infection** |  |  |  |  |  |  | 52.1 |  |  | - |  |  | - | - |  |  |  |  |  | - | - |  |  | - | 2.8 |  | **2.8** |
| **Insufficient Calcium Intake** |  |  |  |  |  | 9.5 |  |  |  | - |  |  | - | - |  |  |  |  |  | - | - |  |  | - |  |  | **1.1** |
| **Chronic H. pylori Infection** |  |  |  | 38.7 |  |  |  |  |  | - |  |  | - | - |  |  |  |  |  | - | - |  |  | - | 2.0 |  | **0.9** |
| **Red Meat Consumption** |  |  |  |  |  | 5.8 |  |  |  | - |  |  | - | - |  |  |  |  |  | - | - |  |  | - |  |  | **0.7** |
| **Chronic HBV Infection** |  |  |  |  |  |  | 24.2 |  |  | - |  |  | - | - |  |  |  |  |  | - | - |  |  | - |  |  | **1.3** |
| **HHV-8 Infection** |  |  |  |  |  |  |  |  |  | - |  |  | - | - |  |  |  |  |  | - | - |  |  | - |  | 100.0 | **0.4** |
| **All Factors** | **87.5** | **77.6** | **66.7** | **58.8** | **28.1** | **59.5** | **83.2** | **47.0** | **51.0** | **-** | **27.3** | **28.0** | **-** | **-** | **22.0** | **3.1** | **17.7** | **23.5** | **20.2** | **-** | **-** | **44.1** | **22.4** | **-** | **4.5** | **100.0** | **38.9** |
| **Women** | | | | | | | | | | | | | | | | | | | | | | | | | | | |
| **Tobacco Smoking** | 87.6 | 64.6 | 58.1 | 17.0 | 22.9 | 16.3 | 19.3 | 8.4 | 51.8 | 0.1 | 4.1 | 28.0 |  |  |  | - |  |  |  |  |  | - |  | 20.2 |  |  | **15.8** |
| **Overweight & Obesity** |  | 15.2 | 5.4 | 2.8 | 11.3 | 5.8 | 22.0 | 27.0 |  | 6.9 |  |  | 11.4 | 42.4 | 24.7 | - | 4.5 | 15.5 | 20.4 |  |  | - |  |  |  |  | **9.8** |
| **Alcohol Consumption** |  | 12.4 | 14.6 | 1.3 |  | 2.0 | 9.8 |  |  |  |  |  | 3.6 |  |  | - |  |  |  |  |  | - |  |  |  |  | **1.8** |
| **Insufficient Physical Activity** |  |  |  |  |  | 11.6 |  |  |  |  |  |  | 3.8 | 24.0 |  | - |  |  |  |  |  | - |  |  |  |  | **3.9** |
| **HPV Infection** |  | 15.9 |  |  |  |  |  |  |  |  |  |  |  |  |  | - |  |  |  | 38.1 | 47.9 | - | 50.4 | 100.0 |  |  | **3.2** |
| **Insufficient Fiber Intake** |  |  |  |  |  | 13.8 |  |  |  |  |  |  |  |  |  | - |  |  |  |  |  | - |  |  |  |  | **1.4** |
| **Processed Meat Consumption** |  |  |  |  |  | 12.7 |  |  |  |  |  |  |  |  |  | - |  |  |  |  |  | - |  |  |  |  | **1.3** |
| **Chronic HCV Infection** |  |  |  |  |  |  | 31.1 |  |  |  |  |  |  |  |  | - |  |  |  |  |  | - |  |  | 2.1 |  | **0.6** |
| **Insufficient Calcium Intake** |  |  |  |  |  | 12.9 |  |  |  |  |  |  |  |  |  | - |  |  |  |  |  | - |  |  |  |  | **1.3** |
| **Chronic H. pylori Infection** |  |  |  | 42.4 |  |  |  |  |  |  |  |  |  |  |  | - |  |  |  |  |  | - |  |  | 0.9 |  | **0.8** |
| **Red Meat Consumption** |  |  |  |  |  | 0.2 |  |  |  |  |  |  |  |  |  | - |  |  |  |  |  | - |  |  |  |  | **0.0** |
| **Chronic HBV Infection** |  |  |  |  |  |  | 11.9 |  |  |  |  |  |  |  |  | - |  |  |  |  |  | - |  |  |  |  | **0.2** |
| **HHV-8 Infection** |  |  |  |  |  |  |  |  |  |  |  |  |  |  |  | - |  |  |  |  |  | - |  |  |  | 100.0 | **0.1** |
| **All Factors** | **87.6** | **77.9** | **66.1** | **54.1** | **31.7** | **55.3** | **65.5** | **33.0** | **51.8** | **7.1** | **4.1** | **28.0** | **17.8** | **56.3** | **24.7** | **-** | **4.5** | **15.5** | **20.4** | **38.1** | **47.9** | **-** | **50.4** | **100.0** | **3.2** | **100.0** | **34.5** |
| **Persons** | | | | | | | | | | | | | | | | | | | | | | | | | | | |
| **Tobacco Smoking** | 87.5 | 66.7 | 54.8 | 23.1 | 20.1 | 15.6 | 34.2 | 19.6 | 51.3 | 0.1 | 16.0 | 28.0 |  |  |  |  |  |  |  |  |  |  |  | 20.2 |  |  | **20.1** |
| **Overweight & Obesity** |  | 14.4 | 7.8 | 2.5 | 12.4 | 7.5 | 21.5 | 27.1 |  | 6.9 |  |  | 11.4 | 42.4 | 23.9 | 3.1 | 7.5 | 19.6 | 20.3 |  |  |  |  |  |  |  | **8.3** |
| **Alcohol Consumption** |  | 13.2 | 19.5 | 3.1 |  | 7.5 | 4.6 |  |  |  |  |  | 3.6 |  |  |  |  |  |  |  |  |  |  |  |  |  | **2.2** |
| **Insufficient Physical Activity** |  |  |  |  |  | 10.3 |  |  |  |  |  |  | 3.8 | 24.0 |  |  |  |  |  |  |  |  |  |  |  |  | **2.5** |
| **HPV Infection** |  | 9.9 |  |  |  |  |  |  |  |  |  |  |  |  |  |  |  |  |  | 38.1 | 47.9 | 44.1 | 31.8 | 100.0 |  |  | **1.9** |
| **Insufficient Fiber Intake** |  |  |  |  |  | 14.3 |  |  |  |  |  |  |  |  |  |  |  |  |  |  |  |  |  |  |  |  | **1.6** |
| **Processed Meat Consumption** |  |  |  |  |  | 11.0 |  |  |  |  |  |  |  |  |  |  |  |  |  |  |  |  |  |  |  |  | **1.2** |
| **Chronic HCV Infection** |  |  |  |  |  |  | 47.0 |  |  |  |  |  |  |  |  |  |  |  |  |  |  |  |  |  | 2.5 |  | **1.7** |
| **Insufficient Calcium Intake** |  |  |  |  |  | 11.1 |  |  |  |  |  |  |  |  |  |  |  |  |  |  |  |  |  |  |  |  | **1.2** |
| **Chronic H. pylori Infection** |  |  |  | 40.4 |  |  |  |  |  |  |  |  |  |  |  |  |  |  |  |  |  |  |  |  | 1.5 |  | **0.9** |
| **Red Meat Consumption** |  |  |  |  |  | 3.2 |  |  |  |  |  |  |  |  |  |  |  |  |  |  |  |  |  |  |  |  | **0.4** |
| **Chronic HBV Infection** |  |  |  |  |  |  | 21.2 |  |  |  |  |  |  |  |  |  |  |  |  |  |  |  |  |  |  |  | **0.7** |
| **HHV-8 Infection** |  |  |  |  |  |  |  |  |  |  |  |  |  |  |  |  |  |  |  |  |  |  |  |  |  | 100.0 | **0.3** |
| **All Factors** | **87.5** | **77.8** | **66.4** | **56.6** | **30.0** | **57.5** | **79.4** | **41.4** | **51.3** | **7.1** | **16.0** | **28.0** | **17.8** | **56.3** | **23.9** | **3.1** | **7.5** | **19.6** | **20.3** | **38.1** | **47.9** | **44.1** | **31.8** | **100.0** | **3.9** | **100.0** | **36.8** |

*Excluding basal cell carcinoma and squamous cell carcinoma of the skin. All cancers combined are displayed as PAF (excess cases).
