# Supplementary material for: Preventable causes of cancer in Texas by race/ethnicity: Major modifiable risk factors in the population
Source: PLoS One. 2022 Oct 13;17(10):e0274905. doi: 10.1371/journal.pone.0274905 (PMC9560474; doi:10.1371/journal.pone.0274905)
Supplement: S11 Table — (DOCX) [file pone.0274905.s018.docx]

**S11 Table.** Age-weighted PAFs of cancers attributable to modifiable risk factors in Texas in 2015 for Hispanics (%), adults aged ≥25 years.

| **Hispanics** | **Lung, Bronchus** | **Mouth, Pharynx, Larynx** | **Esophagus** | **Stomach** | **Pancreas** | **Colorectum** | **Liver** | **Kidney, Renal Pelvis, Ureter** | **Bladder** | **Ovary** | **Myeloid Leukemia** | **Nasal Cavity, Accessory Sinuses** | **Breast** | **Uterus** | **Gallbladder** | **Prostate** | **Thyroid** | **Multiple Myeloma** | **Meningioma** | **Vulva** | **Vagina** | **Penis** | **Anus** | **Cervix** | **NHL** | **Kaposi Sarcoma** | **All Cancers*** |
| --- | --- | --- | --- | --- | --- | --- | --- | --- | --- | --- | --- | --- | --- | --- | --- | --- | --- | --- | --- | --- | --- | --- | --- | --- | --- | --- | --- |
| **Men** | | | | | | | | | | | | | | | | | | | | | | | | | | | |
| **Tobacco Smoking** | 85.8 | 59.4 | 53.3 | 25.8 | 10.0 | 13.1 | 31.3 | 24.7 | 51.0 | - | 23.6 | 24.3 | - | - |  |  |  |  |  | - | - |  |  | - |  |  | **19.6** |
| **Overweight & Obesity** |  | 13.2 | 22.7 | 2.3 | 13.2 | 8.4 | 20.2 | 26.0 |  | - |  |  | - | - | 22.2 | 3.1 | 15.9 | 23.5 | 18.9 | - | - |  |  | - |  |  | **7.7** |
| **Alcohol Consumption** |  | 15.3 | 14.5 | 3.8 |  | 11.8 | 3.7 |  |  | - |  |  | - | - |  |  |  |  |  | - | - |  |  | - |  |  | **2.9** |
| **Insufficient Physical Activity** |  |  |  |  |  | 8.3 |  |  |  | - |  |  | - | - |  |  |  |  |  | - | - |  |  | - |  |  | **1.2** |
| **HPV Infection** |  | 4.4 |  |  |  |  |  |  |  | - |  |  | - | - |  |  |  |  |  | - | - | 21.9 | 11.3 | - |  |  | **0.3** |
| **Insufficient Fiber Intake** |  |  |  |  |  | 11.5 |  |  |  | - |  |  | - | - |  |  |  |  |  | - | - |  |  | - |  |  | **1.6** |
| **Processed Meat Consumption** |  |  |  |  |  | 7.4 |  |  |  | - |  |  | - | - |  |  |  |  |  | - | - |  |  | - |  |  | **1.0** |
| **Chronic HCV Infection** |  |  |  |  |  |  | 35.0 |  |  | - |  |  | - | - |  |  |  |  |  | - | - |  |  | - | 1.6 |  | **2.7** |
| **Insufficient Calcium Intake** |  |  |  |  |  | 9.8 |  |  |  | - |  |  | - | - |  |  |  |  |  | - | - |  |  | - |  |  | **1.4** |
| **Chronic H. pylori Infection** |  |  |  | 38.7 |  |  |  |  |  | - |  |  | - | - |  |  |  |  |  | - | - |  |  | - | 1.6 |  | **1.4** |
| **Red Meat Consumption** |  |  |  |  |  | 5.2 |  |  |  | - |  |  | - | - |  |  |  |  |  | - | - |  |  | - |  |  | **0.7** |
| **Chronic HBV Infection** |  |  |  |  |  |  | 7.4 |  |  | - |  |  | - | - |  |  |  |  |  | - | - |  |  | - |  |  | **0.6** |
| **HHV-8 Infection** |  |  |  |  |  |  |  |  |  | - |  |  | - | - |  |  |  |  |  | - | - |  |  | - |  | 100.0 | **0.3** |
| **All Factors** | **85.8** | **71.4** | **69.0** | **57.3** | **22.0** | **54.9** | **68.2** | **44.3** | **51.0** | **-** | **23.6** | **24.3** | **-** | **-** | **22.2** | **3.1** | **15.9** | **23.5** | **18.9** | **-** | **-** | **21.9** | **11.3** | **-** | **3.2** | **100.0** | **35.6** |
| **Women** | | | | | | | | | | | | | | | | | | | | | | | | | | | |
| **Tobacco Smoking** | 72.0 | 40.2 | 34.7 | 7.7 | 9.4 | 8.2 | 8.3 | 4.6 | 27.0 | 0.1 | 8.5 | 14.9 |  |  |  | - |  |  |  |  |  | - |  | 11.6 |  |  | **6.8** |
| **Overweight & Obesity** |  | 13.9 | 10.8 | 1.8 | 10.2 | 5.3 | 20.4 | 24.7 |  | 6.1 |  |  | 10.5 | 38.0 | 24.1 | - | 3.8 | 13.9 | 17.6 |  |  | - |  |  |  |  | **9.8** |
| **Alcohol Consumption** |  | 27.0 | 12.3 | 1.1 |  | 2.5 | 17.9 |  |  |  |  |  | 6.6 |  |  | - |  |  |  |  |  | - |  |  |  |  | **3.0** |
| **Insufficient Physical Activity** |  |  |  |  |  | 10.8 |  |  |  |  |  |  | 3.8 | 22.7 |  | - |  |  |  |  |  | - |  |  |  |  | **3.9** |
| **HPV Infection** |  | 9.0 |  |  |  |  |  |  |  |  |  |  |  |  |  | - |  |  |  | 17.5 | 32.6 | - | 29.9 | 100.0 |  |  | **4.7** |
| **Insufficient Fiber Intake** |  |  |  |  |  | 11.6 |  |  |  |  |  |  |  |  |  | - |  |  |  |  |  | - |  |  |  |  | **1.0** |
| **Processed Meat Consumption** |  |  |  |  |  | 10.4 |  |  |  |  |  |  |  |  |  | - |  |  |  |  |  | - |  |  |  |  | **0.9** |
| **Chronic HCV Infection** |  |  |  |  |  |  | 4.7 |  |  |  |  |  |  |  |  | - |  |  |  |  |  | - |  |  | 0.2 |  | **0.1** |
| **Insufficient Calcium Intake** |  |  |  |  |  | 10.2 |  |  |  |  |  |  |  |  |  | - |  |  |  |  |  | - |  |  |  |  | **0.9** |
| **Chronic H. pylori Infection** |  |  |  | 38.8 |  |  |  |  |  |  |  |  |  |  |  | - |  |  |  |  |  | - |  |  | 1.2 |  | **1.0** |
| **Red Meat Consumption** |  |  |  |  |  | 0.3 |  |  |  |  |  |  |  |  |  | - |  |  |  |  |  | - |  |  |  |  | **0.0** |
| **Chronic HBV Infection** |  |  |  |  |  |  | 0.0 |  |  |  |  |  |  |  |  | - |  |  |  |  |  | - |  |  |  |  | **0.0** |
| **HHV-8 Infection** |  |  |  |  |  |  |  |  |  |  |  |  |  |  |  | - |  |  |  |  |  | - |  |  |  | 100.0 | **0.0** |
| **All Factors** | **72.0** | **65.6** | **48.7** | **45.3** | **18.7** | **46.4** | **42.9** | **28.1** | **27.0** | **6.2** | **8.5** | **14.9** | **19.6** | **52.1** | **24.1** | **-** | **3.8** | **13.9** | **17.6** | **17.5** | **32.6** | **-** | **29.9** | **100.0** | **1.5** | **100.0** | **28.2** |
| **Persons** | | | | | | | | | | | | | | | | | | | | | | | | | | | |
| **Tobacco Smoking** | 80.1 | 55.0 | 50.3 | 17.7 | 9.8 | 11.1 | 25.0 | 16.4 | 45.1 | 0.1 | 17.5 | 22.3 |  |  |  |  |  |  |  |  |  |  |  | 11.6 |  |  | **12.8** |
| **Overweight & Obesity** |  | 13.3 | 20.8 | 2.1 | 11.8 | 7.1 | 20.2 | 25.4 |  | 6.1 |  |  | 10.5 | 38.0 | 23.6 | 3.1 | 6.2 | 19.1 | 18.2 |  |  |  |  |  |  |  | **8.8** |
| **Alcohol Consumption** |  | 18.0 | 14.2 | 2.6 |  | 8.0 | 7.6 |  |  |  |  |  | 6.6 |  |  |  |  |  |  |  |  |  |  |  |  |  | **3.0** |
| **Insufficient Physical Activity** |  |  |  |  |  | 9.3 |  |  |  |  |  |  | 3.8 | 22.7 |  |  |  |  |  |  |  |  |  |  |  |  | **2.6** |
| **HPV Infection** |  | 5.5 |  |  |  |  |  |  |  |  |  |  |  |  |  |  |  |  |  | 17.5 | 32.6 | 21.9 | 22.6 | 100.0 |  |  | **2.6** |
| **Insufficient Fiber Intake** |  |  |  |  |  | 11.6 |  |  |  |  |  |  |  |  |  |  |  |  |  |  |  |  |  |  |  |  | **1.3** |
| **Processed Meat Consumption** |  |  |  |  |  | 8.7 |  |  |  |  |  |  |  |  |  |  |  |  |  |  |  |  |  |  |  |  | **1.0** |
| **Chronic HCV Infection** |  |  |  |  |  |  | 26.6 |  |  |  |  |  |  |  |  |  |  |  |  |  |  |  |  |  | 1.0 |  | **1.4** |
| **Insufficient Calcium Intake** |  |  |  |  |  | 10.0 |  |  |  |  |  |  |  |  |  |  |  |  |  |  |  |  |  |  |  |  | **1.1** |
| **Chronic H. pylori Infection** |  |  |  | 38.8 |  |  |  |  |  |  |  |  |  |  |  |  |  |  |  |  |  |  |  |  | 1.4 |  | **1.1** |
| **Red Meat Consumption** |  |  |  |  |  | 3.2 |  |  |  |  |  |  |  |  |  |  |  |  |  |  |  |  |  |  |  |  | **0.4** |
| **Chronic HBV Infection** |  |  |  |  |  |  | 5.3 |  |  |  |  |  |  |  |  |  |  |  |  |  |  |  |  |  |  |  | **0.3** |
| **HHV-8 Infection** |  |  |  |  |  |  |  |  |  |  |  |  |  |  |  |  |  |  |  |  |  |  |  |  |  | 100.0 | **0.2** |
| **All Factors** | **80.1** | **69.8** | **66.4** | **52.0** | **20.4** | **51.5** | **61.6** | **37.6** | **45.1** | **6.2** | **17.5** | **22.3** | **19.6** | **52.1** | **23.6** | **3.1** | **6.2** | **19.1** | **18.2** | **17.5** | **32.6** | **21.9** | **22.6** | **100.0** | **2.5** | **100.0** | **31.7** |

*Excluding basal cell carcinoma and squamous cell carcinoma of the skin. All cancers combined are displayed as PAF (excess cases).
