# Supplementary material for: Preventable causes of cancer in Texas by race/ethnicity: Major modifiable risk factors in the population
Source: PLoS One. 2022 Oct 13;17(10):e0274905. doi: 10.1371/journal.pone.0274905 (PMC9560474; doi:10.1371/journal.pone.0274905)
Supplement: S12 Table — (DOCX) [file pone.0274905.s019.docx]

**S12 Table.** Age-weighted PAFs of cancers attributable to modifiable risk factors in Texas in 2015 for Other Races/Ethnicities (%), adults aged ≥25 years.

| **Other Races/Ethnicities** | **Lung, Bronchus** | **Mouth, Pharynx, Larynx** | **Esophagus** | **Stomach** | **Pancreas** | **Colorectum** | **Liver** | **Kidney, Renal Pelvis, Ureter** | **Bladder** | **Ovary** | **Myeloid Leukemia** | **Nasal Cavity, Accessory Sinuses** | **Breast** | **Uterus** | **Gallbladder** | **Prostate** | **Thyroid** | **Multiple Myeloma** | **Meningioma** | **Vulva** | **Vagina** | **Penis** | **Anus** | **Cervix** | **NHL** | **Kaposi Sarcoma** | **All Cancers*** |
| --- | --- | --- | --- | --- | --- | --- | --- | --- | --- | --- | --- | --- | --- | --- | --- | --- | --- | --- | --- | --- | --- | --- | --- | --- | --- | --- | --- |
| **Men** | | | | | | | | | | | | | | | | | | | | | | | | | | | |
| **Tobacco Smoking** | 83.2 | 52.8 | 47.3 | 22.3 | 9.3 | 11.9 | 27.9 | 22.0 | 43.9 | - | 21.3 | 20.7 | - | - |  |  |  |  |  | - | - |  |  | - |  |  | **17.7** |
| **Overweight & Obesity** |  | 14.1 | 17.2 | 2.2 | 13.5 | 8.5 | 21.5 | 24.6 |  | - |  |  | - | - | 22.0 | 2.3 | 15.3 | 24.4 | 0.0 | - | - |  |  | - |  |  | **5.6** |
| **Alcohol Consumption** |  | 3.2 | 1.4 | 0.8 |  | 1.7 | 0.6 |  |  | - |  |  | - | - |  |  |  |  |  | - | - |  |  | - |  |  | **0.4** |
| **Insufficient Physical Activity** |  |  |  |  |  | 7.1 |  |  |  | - |  |  | - | - |  |  |  |  |  | - | - |  |  | - |  |  | **0.6** |
| **HPV Infection** |  | 4.9 |  |  |  |  |  |  |  | - |  |  | - | - |  |  |  |  |  | - | - | 0.0 | 36.6 | - |  |  | **0.3** |
| **Insufficient Fiber Intake** |  |  |  |  |  | 12.4 |  |  |  | - |  |  | - | - |  |  |  |  |  | - | - |  |  | - |  |  | **1.1** |
| **Processed Meat Consumption** |  |  |  |  |  | 4.0 |  |  |  | - |  |  | - | - |  |  |  |  |  | - | - |  |  | - |  |  | **0.4** |
| **Chronic HCV Infection** |  |  |  |  |  |  | 0.0 |  |  | - |  |  | - | - |  |  |  |  |  | - | - |  |  | - | 0.0 |  | **0.0** |
| **Insufficient Calcium Intake** |  |  |  |  |  | 7.2 |  |  |  | - |  |  | - | - |  |  |  |  |  | - | - |  |  | - |  |  | **0.6** |
| **Chronic H. pylori Infection** |  |  |  | 43.3 |  |  |  |  |  | - |  |  | - | - |  |  |  |  |  | - | - |  |  | - | 1.9 |  | **1.4** |
| **Red Meat Consumption** |  |  |  |  |  | 5.3 |  |  |  | - |  |  | - | - |  |  |  |  |  | - | - |  |  | - |  |  | **0.5** |
| **Chronic HBV Infection** |  |  |  |  |  |  | 24.8 |  |  | - |  |  | - | - |  |  |  |  |  | - | - |  |  | - |  |  | **1.5** |
| **HHV-8 Infection** |  |  |  |  |  |  |  |  |  | - |  |  | - | - |  |  |  |  |  | - | - |  |  | - |  | 100.0 | **0.3** |
| **All Factors** | **83.2** | **63.0** | **57.4** | **57.8** | **21.7** | **45.4** | **57.6** | **40.8** | **43.9** | **-** | **21.3** | **20.7** | **-** | **-** | **22.0** | **2.3** | **15.3** | **24.4** | **0.0** | **-** | **-** | **0.0** | **36.6** | **-** | **2.4** | **100.0** | **27.7** |
| **Women** | | | | | | | | | | | | | | | | | | | | | | | | | | | |
| **Tobacco Smoking** | 78.1 | 48.5 | 38.5 | 11.6 | 14.0 | 12.3 | 11.5 | 6.5 | 34.6 | 0.5 | 3.1 | 15.8 |  |  |  | - |  |  |  |  |  | - |  | 21.0 |  |  | **9.5** |
| **Overweight & Obesity** |  | 7.1 | 13.9 | 2.3 | 6.0 | 3.0 | 12.3 | 16.8 |  | 3.4 |  |  | 5.3 | 26.2 | 13.2 | - | 2.3 | 8.5 | 0.0 |  |  | - |  |  |  |  | **5.1** |
| **Alcohol Consumption** |  | 6.2 | 6.5 | 0.5 |  | 1.0 | 5.7 |  |  |  |  |  | 2.4 |  |  | - |  |  |  |  |  | - |  |  |  |  | **1.2** |
| **Insufficient Physical Activity** |  |  |  |  |  | 7.8 |  |  |  |  |  |  | 4.2 | 20.8 |  | - |  |  |  |  |  | - |  |  |  |  | **3.4** |
| **HPV Infection** |  | 7.9 |  |  |  |  |  |  |  |  |  |  |  |  |  | - |  |  |  | 8.1 | 27.0 | - | 0.0 | 100.0 |  |  | **2.8** |
| **Insufficient Fiber Intake** |  |  |  |  |  | 11.4 |  |  |  |  |  |  |  |  |  | - |  |  |  |  |  | - |  |  |  |  | **1.0** |
| **Processed Meat Consumption** |  |  |  |  |  | 6.6 |  |  |  |  |  |  |  |  |  | - |  |  |  |  |  | - |  |  |  |  | **0.5** |
| **Chronic HCV Infection** |  |  |  |  |  |  | 15.1 |  |  |  |  |  |  |  |  | - |  |  |  |  |  | - |  |  | 2.4 |  | **0.3** |
| **Insufficient Calcium Intake** |  |  |  |  |  | 10.9 |  |  |  |  |  |  |  |  |  | - |  |  |  |  |  | - |  |  |  |  | **0.9** |
| **Chronic H. pylori Infection** |  |  |  | 41.9 |  |  |  |  |  |  |  |  |  |  |  | - |  |  |  |  |  | - |  |  | 0.0 |  | **0.8** |
| **Red Meat Consumption** |  |  |  |  |  | 0.1 |  |  |  |  |  |  |  |  |  | - |  |  |  |  |  | - |  |  |  |  | **0.0** |
| **Chronic HBV Infection** |  |  |  |  |  |  | 0.7 |  |  |  |  |  |  |  |  | - |  |  |  |  |  | - |  |  |  |  | **0.0** |
| **HHV-8 Infection** |  |  |  |  |  |  |  |  |  |  |  |  |  |  |  | - |  |  |  |  |  | - |  |  |  | 100.0 | **0.0** |
| **All Factors** | **78.1** | **57.8** | **59.7** | **49.9** | **19.2** | **42.9** | **38.7** | **22.1** | **34.6** | **3.9** | **3.1** | **15.8** | **11.6** | **41.3** | **13.2** | **-** | **2.3** | **8.5** | **0.0** | **8.1** | **27.0** | **-** | **0.0** | **100.0** | **1.8** | **100.0** | **23.1** |
| **Persons** | | | | | | | | | | | | | | | | | | | | | | | | | | | |
| **Tobacco Smoking** | 81.1 | 51.4 | 46.0 | 17.9 | 11.8 | 12.1 | 24.0 | 16.0 | 42.2 | 0.5 | 11.6 | 18.9 |  |  |  |  |  |  |  |  |  |  |  | 21.0 |  |  | **13.4** |
| **Overweight & Obesity** |  | 11.8 | 16.7 | 2.2 | 9.5 | 5.7 | 19.3 | 21.6 |  | 3.4 |  |  | 5.3 | 26.2 | 17.0 | 2.3 | 5.5 | 16.5 | 0.0 |  |  |  |  |  |  |  | **5.4** |
| **Alcohol Consumption** |  | 4.2 | 2.1 | 0.7 |  | 1.4 | 1.8 |  |  |  |  |  | 2.4 |  |  |  |  |  |  |  |  |  |  |  |  |  | **0.8** |
| **Insufficient Physical Activity** |  |  |  |  |  | 7.4 |  |  |  |  |  |  | 4.2 | 20.8 |  |  |  |  |  |  |  |  |  |  |  |  | **2.1** |
| **HPV Infection** |  | 5.9 |  |  |  |  |  |  |  |  |  |  |  |  |  |  |  |  |  | 8.1 | 27.0 | 0.0 | 36.6 | 100.0 |  |  | **1.6** |
| **Insufficient Fiber Intake** |  |  |  |  |  | 11.9 |  |  |  |  |  |  |  |  |  |  |  |  |  |  |  |  |  |  |  |  | **1.0** |
| **Processed Meat Consumption** |  |  |  |  |  | 5.3 |  |  |  |  |  |  |  |  |  |  |  |  |  |  |  |  |  |  |  |  | **0.5** |
| **Chronic HCV Infection** |  |  |  |  |  |  | 3.6 |  |  |  |  |  |  |  |  |  |  |  |  |  |  |  |  |  | 1.0 |  | **0.2** |
| **Insufficient Calcium Intake** |  |  |  |  |  | 9.1 |  |  |  |  |  |  |  |  |  |  |  |  |  |  |  |  |  |  |  |  | **0.8** |
| **Chronic H. pylori Infection** |  |  |  | 42.7 |  |  |  |  |  |  |  |  |  |  |  |  |  |  |  |  |  |  |  |  | 1.1 |  | **1.1** |
| **Red Meat Consumption** |  |  |  |  |  | 2.6 |  |  |  |  |  |  |  |  |  |  |  |  |  |  |  |  |  |  |  |  | **0.2** |
| **Chronic HBV Infection** |  |  |  |  |  |  | 19.0 |  |  |  |  |  |  |  |  |  |  |  |  |  |  |  |  |  |  |  | **0.7** |
| **HHV-8 Infection** |  |  |  |  |  |  |  |  |  |  |  |  |  |  |  |  |  |  |  |  |  |  |  |  |  | 100.0 | **0.1** |
| **All Factors** | **81.1** | **61.3** | **54.7** | **54.6** | **20.4** | **44.0** | **53.0** | **33.9** | **42.2** | **3.9** | **11.6** | **18.9** | **11.6** | **41.3** | **17.0** | **2.3** | **5.5** | **16.5** | **0.0** | **8.1** | **27.0** | **0.0** | **36.6** | **100.0** | **2.4** | **100.0** | **25.3** |

*Excluding basal cell carcinoma and squamous cell carcinoma of the skin. All cancers combined are displayed as PAF (excess cases).

**Reference list**

1. World Cancer Research Fund/American Institute for Cancer Research. Continuous Update Project Expert Report 2018. Alcoholic drinks and the risk of cancer. Available at dietandcancerreport.org.
2. Carter BD, Abnet CC, Feskanich D, et al. Smoking and mortality - beyond established causes. N Engl J Med. 2015;372:631-40. doi:10.1056/NEJMsa1407211.
3. Ismali F, Goding Sauer A, Miller KD, et al. Proportion and Number of Cancer Cases and Deaths Attributable to Potentially Modifiable Risk Factors in the United States. CA Cancer J Clin. 2018;68:31-54. doi:10.3322/caac.21440.
4. Roura E, Castellsague C, Pawlita M, et al. Smoking as a major risk factor for cervical cancer and pre-cancer: results from the EPIC cohort. Int J Cancer. 2014;135(2):453-66. doi:10.1002/ijc.28666.
5. World Cancer Research Fund/American Institute for Cancer Research. Continuous Update Project Expert Report 2018. Meat, fish and dairy products and the risk of cancer. Available at dietandcancerreport.org.
6. World Cancer Research Fund/American Institute for Cancer Research. Continuous Update Project Expert Report 2018. Wholegrains, vegetables and fruit and the risk of cancer. Available at dietandcancerreport.org
7. Plummer M, de Martel C, Vignat J, Ferlay J, Bray F, Franceschi S. Global burden of cancers attributable to infections in 2012: a synthetic analysis. Lancet Glob Health. 2016;4(9):e609-616. doi:10.1016/S2214-109X(16)30143-7.
8. de Martel C, Ferlay J, Franceschi S, et al. Global burden of cancers attributable to infections in 2008: a review and synthetic analysis. Lancet Oncol. 2012;13(6):607-615. doi:10.1016/S1470-2045(12)70137-7.
9. Carter JJ, Madeleine MM, Shera K, et al. Human papillomavirus 16 and 18 L1 serology compared across anogenital cancer sites. Cancer Res. 2001;61(5):1934-1940.
10. Anantharaman D, Gheit T, Waterboer T, et al. Human papillomavirus infections and upper aero-digestive tract cancers: the ARCAGE Study. J Natl Cancer Inst. 2013;105(8):536-545. doi:10.1093/jnci/djt053.
11. World Cancer Research Fund/American Institute for Cancer Research. Continuous Update Project Expert Report 2018. Body fatness and weight gain and the risk of cancer. Available at dietandcancerreport.org.
12. World Cancer Research Fund/American Institute for Cancer Research. Continuous Update Project: World Cancer Research Fund International Systematic Literature Review 2015. The Associations between Food, Nutrition, and Physical Activity and the Risk of Stomach Cancer. Available at dietandcancerreport.org.
13. Kitahara CM, McCullough ML, Franceschi S, et al. Anthropometric Factors and Thyroid Cancer Risk by Histological Subtype: Pooled Analysis of 22 Prospective Studies. Thyroid. 2016 Feb;26(2):306-18. doi: 10.1089/thy.2015.0319.
14. IARC. Absence of excess body fatness. IARC Handb Cancer Prev. 2018;16:1–646. Available from: <http://publications.iarc.fr/570>.
15. Marinac CR, Birmann BM, Lee IM, et al. Body mass index through adulthood, physical activity, and risk of multiple myeloma: a prospective analysis in three large cohorts. Br J Cancer. 2018;118(7):1013-1019. doi: 10.1038/s41416-018-0010-4.
16. Zhang D, Chen J, Wang J, et al. Body mass index and risk of brain tumors: a systematic review and dose-response meta-analysis. Eur J Clin Nutr. 2016;70(7):757-65. doi: 10.1038/ejcn.2016.4
