# Supplementary material for: Preventable causes of cancer in Texas by race/ethnicity: Major modifiable risk factors in the population
Source: PLoS One. 2022 Oct 13;17(10):e0274905. doi: 10.1371/journal.pone.0274905 (PMC9560474; doi:10.1371/journal.pone.0274905)
Supplement: S1 File — (DOCX) [file pone.0274905.s020.docx]

**SUPPLEMENTARY MATERIALS**

**METHODS**

Tobacco smoking was categorized by current smokers (those who had smoked 100 cigarettes in their lifetime and were currently smoking some days or every day) and former smokers (those who had smoked 100 cigarettes in their lifetime and were not currently smoking).^1^ Body mass index (BMI) was used to classify overweight (BMI 25-<30 kg/m^2^) and obesity (BMI ≥30 kg/m^2^). Alcohol consumption was categorized by 7 exposure categories of number of drinks consumed per day (0 drinks/day, 0<drinks/day≤1, 1<drinks/day≤2, 2<drinks/day≤3, 3<drinks/day≤4, 4<drinks/day≤5, drinks/day>5), with standard drink size defined by the U.S. Department of Health and Human Services’ and U.S. Department of Agriculture’s *2015-2020* *Dietary Guidelines for Americans*.^2^ Insufficient physical activity was measured by deficit from reference levels in metabolic equivalents of task (MET)-minutes per week, with 1000 MET-minutes/week serving as the reference level for combined moderate- or vigorous-intensity exercise and 500 MET-minutes/week as the reference level for only vigorous-intensity exercise, in accordance with prior studies^3^ and based on the U.S. Department of Health and Human Services’ *Physical Activity Guidelines for American;*^4^ physical activity levels were classified into categories of moderate- or vigorous-intensity activity (750-999 MET-min/wk, 500-749 MET-min/wk, 250-499 MET-min/wk, 0-249 MET-min/wk) and only vigorous-intensity activity (250-499 MET-min/wk, 0-249 MET-min/wk).^3^ Infection prevalence was determined by seropositivity to relevant infection markers (chronic HBV: positive hepatitis B surface antigen;^5^ chronic HCV: positive or indeterminate anti-HCV and positive HCV RNA;^3^ HPV: positive 9-plex Competitive Luminex Assay for L1-viral like particles;^6^ *H. pylori*: positive anti-*H. pylori* IgG;^7^ HHV-8: prevalence data not collected as the PAF is assumed to be 100%^8^). Meat consumption was categorized by consumption in excess of reference levels (red meat: reference ≤60 grams/day, categories [≤60 grams/day, 60-80 grams/day, 80-100 grams/day, 100-120 grams/day, 120-140 grams/day, >140 grams/day]; processed meat: reference 0 grams/day, categories [0 grams/day, 0-35 grams/day, 35-70 grams/day, 70-105 grams/day, 105-140 grams/day, >140 grams/day]).^9^ Fiber and calcium intake were classified by categories of deficit from reference levels (fiber: reference ≥28 grams/day, categories [≥28 grams/day, 21-28 grams/day, 14-21 grams/day, 7-14 grams/day, 0-7 grams/day]; calcium: reference ≥1000 milligrams/day, categories [≥1000 mg/day, 800-1000 mg/day, 600-800 mg/day, 400-600 mg/day, 200-400 mg/day, 0-200 mg/day]).^2^ For HPV infection and meat consumption, the oldest two age groups were set at 65-69 and ≥70 years due to limitations in prevalence data.

**REFERENCES**

1. Texas Behavioral Risk Factor Surveillance System (BRFSS) Data Visualizations – Selected Risk Factors. Texas Department of State Health Services Website. http://healthdata.dshs.texas.gov/CommunitySurveys/BRFSS. Accessed March 28, 2019.
2. U.S. Department of Health and Human Services and U.S. Department of Agriculture. *2015-2020 Dietary Guidelines for Americans*. 8^th^ Edition. December 2015. Available at https://health.gov/dietaryguidelines/2015/guidelines/.
3. Islami F, Goding Sauer A, Miller KD, et al. Proportion and number of cancer cases and deaths attributable to potentially modifiable risk factors in the United States. *CA Cancer J Clin*. 2018;68(1):31-54. doi: 10.3322.caac.21440
4. U.S. Department of Health and Human Services. *Physical Activity Guidelines for Americans, 2nd edition*. Washington, DC: U.S. Department of Health and Human Services; 2018.
5. Seto WK, Lo YR, Pawlotsky JM, Yuen MF. Chronic hepatitis B virus infection. *Lancet*. 2018;392(10161):P2313-2324. doi:10.1016/S0140-6736(18)31865-8.
6. Centers for Disease Control and Prevention (CDC). National Center for Health Statistics (NCHS). National Health and Nutrition Examination Survey Data. Hyattsville, MD: U.S. Department of Health and Human Services, Centers for Disease Control and Prevention, 2005-2006. https://wwwn.cdc.gov/nchs/nhanes/search/datapage.aspx?Component=Laboratory&CycleBeginYear=2005.
7. Centers for Disease Control and Prevention (CDC). National Center for Health Statistics (NCHS). National Health and Nutrition Examination Survey Data. Hyattsville, MD: U.S. Department of Health and Human Services, Centers for Disease Control and Prevention, 1999-2000. https://wwwn.cdc.gov/Nchs/Nhanes/Search/DataPage.aspx?Component=Laboratory&CycleBeginYear=1999.
8. Plummer M, de Martel C, Vignat J, Ferlay J, Bray F, Franceschi S. Global burden of cancers attributable to infections in 2012: a synthetic analysis. *Lancet Glob Health.* 2016;4:e609-16. doi: 10.1016/ S2214-109X(16)30143-7
9. World Cancer Research Fund/American Institute for Cancer Research. *Diet, Nutrition, Physical Activity and Cancer: a Global Perspective*. Continuous Update Project Expert Report 2018. Available at dietandcancerreport.org.
